# Whole genome duplication events in plant evolution reconstructed and predicted using myosin motor proteins

**DOI:** 10.1186/1471-2148-13-202

**Published:** 2013-09-22

**Authors:** Stefanie Mühlhausen, Martin Kollmar

**Affiliations:** 1 Group Systems Biology of Motor Proteins, Department of NMR-based Structural Biology, Max-Planck-Institute for biophysical Chemistry, Göttingen, Germany

**Keywords:** Myosin, Plant evolution, Whole genome duplication

## Abstract

**Background:**

The evolution of land plants is characterized by whole genome duplications (WGD), which drove species diversification and evolutionary novelties. Detecting these events is especially difficult if they date back to the origin of the plant kingdom. Established methods for reconstructing WGDs include intra- and inter-genome comparisons, *K*_S_ age distribution analyses, and phylogenetic tree constructions.

**Results:**

By analysing 67 completely sequenced plant genomes 775 myosins were identified and manually assembled. Phylogenetic trees of the myosin motor domains revealed orthologous and paralogous relationships and were consistent with recent species trees. Based on the myosin inventories and the phylogenetic trees, we have identified duplications of the entire myosin motor protein family at timings consistent with 23 WGDs, that had been reported before. We also predict 6 WGDs based on further protein family duplications. Notably, the myosin data support the two recently reported WGDs in the common ancestor of all extant angiosperms. We predict single WGDs in the *Manihot esculenta* and *Nicotiana benthamiana* lineages, two WGDs for *Linum usitatissimum* and *Phoenix dactylifera*, and a triplication or two WGDs for *Gossypium raimondii*. Our data show another myosin duplication in the ancestor of the angiosperms that could be either the result of a single gene duplication or a remnant of a WGD.

**Conclusions:**

We have shown that the myosin inventories in angiosperms retain evidence of numerous WGDs that happened throughout plant evolution. In contrast to other protein families, many myosins are still present in extant species. They are closely related and have similar domain architectures, and their phylogenetic grouping follows the genome duplications. Because of its broad taxonomic sampling the dataset provides the basis for reliable future identification of further whole genome duplications.

## Background

Whole genome duplications have had a strong impact on species diversification and may have triggered evolutionary novelties [1,2]. Plants underwent several independent rounds of whole genome duplication (WGD) events [3-9]. Traces of these WGDs are still present, although duplication events are usually followed by massive gene loss and structural rearrangements [10]. Nevertheless, many cases of both recent and ancient WGD events have been reported so far, including the hexaploidy event shared by most, if not all, eudicots [11-13], and WGDs dated to the common ancestor of all extant angiosperms and to the common ancestor of all extant seed plants [14].

Whole genome duplications are usually reconstructed by intra- and inter-genome comparisons to detect synthenic regions (genomic collinearity), by *K*_S_ age distribution analyses, and by phylogenetic tree constructions [15]. Since collinearity decreases with time, it can usually not be used to detect old genome duplications. *K*_S_ describes the number of synonymous substitutions per synonymous site and becomes unreliable in age distribution analyses due to gene loss and saturation effects. Phylogenetic approaches have the advantage that duplication events can be mapped onto gene trees provided that these trees include paralogs created by given WGD events and orthologous genes from other species. However, individual gene trees can be affected by different evolutionary rates of genes between species, pseudogenization and individual gene duplication and loss. To overcome these difficulties, a multigene approach has been undertaken to differentiate between a shared or species-specific WGD in the legumes *Glycine max* and *Medicago truncatula*[16] and a phylogenomics approach to correctly date proposed WGDs early in plant evolution [14]. Nevertheless, for the fast and convenient detection and dating of so far undiscovered WGDs it would be ideal to have a protein family whose evolution has not been affected by the described problems. The difficulty is to identify such a protein family because most genes in plants exist in only one or two copies per genome (e.g. TEL genes [17], CAP and ARP2/3 proteins [18]) while other families like the expansin superfamily and the MADS-box transcription factor genes might contain dozens to over hundred gene family members [19,20].

Myosins constitute one of the largest and most diverse protein families in eukaryotes [21]. They are characterized by a motor domain that binds to actin in an ATP-dependent manner, a neck domain consisting of varying numbers of IQ motifs that each bind either a myosin-specific light chain or a calmodulin or calmodulin-like protein, and amino-terminal and carboxy-terminal domains of various length and function [22]. Myosins are typically classified based on phylogenetic analyses of their motor domains. An analysis of all myosin genes available in 2007 allowed grouping them into 35 classes [23]. While metazoans, fungi and protozoans contain myosins of many different classes, only myosins of class VIII and class XI, are present in and unique for plants. The formerly algae-specific class XIII myosins have been shown to be part of the class XI [23]. Class VIII myosins contain long N-terminal extensions, that have not been characterised in detail so far, and C-terminal coiled-coil regions. Class XI myosins have six IQ motifs followed by an extended coiled-coil region and a DIL domain and thus have domain architectures identical to class V myosins.

Assembling and annotating plant myosins is a continuous effort of our group. Since the major myosin sequence analysis was published in 2007 [23], every newly assembled plant genome had been analysed. Annotated myosin sequences were made available to the community via CyMoBase [24,25]. Since only a few plant genomes had been sequenced in 2007 [23,26] we did not develop a concise nomenclature for the many homologs within the two plant myosin classes. Such a nomenclature should account for whole genome and single gene duplications and thus would require a broad taxonomic sampling. The first plant myosins identified in *Arabidopsis thaliana* had been named ATM1/ATM2 [27,28] and MYA1/MYA2/MYA3 [28,29]. Their recently suggested renaming [30], however, resulted in a mixture of numbers and letters to distinguish class VIII and class XI orthologs and paralogs in order to partly keep the earlier naming of the other 13 *Arabidopsis* myosins [31]. Thus, a comprehensive naming scheme is still missing that would also be flexible enough to incorporate the myosins from the upcoming sequencing projects.

Here, we used the myosin protein family for reconstructing and predicting of WGDs in plant evolution. Myosins represent an outstanding case because in each extant plant species many homologs are present for which unambiguous paralog and ortholog relationships can be reconstructed. We present an analysis of 67 completely sequenced plant species that provides the framework for the identification and placement of WGDs in so far uncovered branches of the plant tree.

## Results

### Identification and annotation of the plant myosins

The genomic regions containing putative myosin genes were identified using *Arabidopsis thaliana* myosins as queries for TBLASTN searches. The protein sequences were then assembled and annotated using *ab initio* gene prediction and cross-species gene reconstruction software followed by manual refinement. For *ab initio* gene predictions we used AUGUSTUS [32] and Genscan [33]. Compared to myosins of other major eukaryotic branches the myosins of plants are relatively conserved and belong to only two classes, class VIII and class XI. As more and more draft genome assemblies of species closely related to already sequenced species become available, known gene annotations can be used as starting point for gene predictions. Here, we used the cross-species search function implemented in the gene reconstruction software WebScipio [34] to obtain myosins from such species. An example is the myosin protein family of *Eutrema halophilum*, which was annotated based on the preceding annotation of the myosins from *Eutrema parvulum.* Manual refinement of *ab initio* predicted and cross-species reconstructed sequences includes correcting wrongly predicted sequence regions, resolving sequencing problems and assembling myosins spread on several contigs. In detail, the comparison of a newly added myosin sequence with already annotated plant myosins in a structure guided, manually refined multiple sequence alignment allowed us to identify missing regions, whose sequences were added by manually inspecting the respective genomic regions, and to delete extra sequence, which has obviously been mis-predicted as exonic region within actually intronic sequence. Notably, plant myosins contain several very short exons that were missing in almost all *ab initio* predictions. During manual refinement we also accounted for in-frame stop codons and frame shifts as result of for example local low-coverage within genomic sequences.

WebScipio has also been used to reconstruct the gene structures of all plant myosins. Through comparison of intron positions and splice-site phases relative to the multiple protein sequence alignments, several suspicious exon borders could be resolved in the less conserved parts of the C-terminal tail regions. Unfortunately, full-length cDNA sequences are only available for about a dozen plant myosins, covering not even all *Arabidopsis thaliana* and *Oryza sativa* myosins. However, the available plant EST and cDNA read data helped in determining for example the correct N-termini of the headless class XI myosins and the C-termini of the short class XI myosins (see below). Plant genomes have been sequenced with different methods (Sanger, Roche/454, Illumina, and combinations of them) and different coverage. Only a few have undergone refinement and extensive closing of assembly gaps. Because myosins are large proteins we only used those genomes in which we could unambiguously reconstruct all myosins. Thus, we excluded the fragmented draft genomes of some species from our analysis. Among these are *Penstemon cyananthus*, *Amaranthus tuberculatus*, *Lotus japonicus*, *Vigna radiate* and *Leersia perrieri*. Nevertheless, some myosin genes contain smaller or larger gaps in many plant genomes. Sequences for which only a small part is missing (up to 5% of the average protein length) were termed “Partials”. “Partials” are not expected to considerably influence the phylogenetic tree computations and were used together with complete sequences for these computations. Sequences with gaps accounting for more than 5% of the expected sequence length were termed “Fragments”. “Fragments” are important for the qualitative analysis to denote the presence of this specific myosin subtype in the respective species but were not used in phylogenetic tree computations because of the long gaps in the alignment. Regions with gaps cannot be excluded from the alignment for tree computations, because the gaps in the “Fragments” are not at the same positions. However, separately adding each single “Fragment” to the alignment and calculating independent trees can unambiguously classify “Fragments”. For instance, a class XI myosin sequence containing about 1,300 residues of the putative 1,560 residues of the full-length sequence would be denoted as “Fragment” but its subtype relationship could be resolved unambiguously. The classification of all annotated myosins from the 67 completely sequenced plants into these three categories based on their respective sequence length is listed in Additional file 1.

The plant myosin dataset contains 828 sequences from 87 plant species. Out of these, 694 motor domain sequences from 67 species are complete and were used in the phylogenetic tree reconstructions. Additionally, phylogenetic trees were calculated based on reduced datasets comprising 380 myosin full length and 221 myosin motor domain sequences of less than 90% identity, respectively. The genome assemblies of *Hordeum vulgare*, *Beta vulgaris*, *Betula nana* (this genome assembly is highly contaminated with DNA from various fungi), *Pyrus x bretschneideri*, and *Jatropha curcas* were made available shortly after we had finished our analysis. Therefore, their myosins were not included in the tree computations but added to the qualitative analysis as examples for easily revealing WGDs in newly sequenced genomes. We tried to identify alternative splice variants based on the extensive cDNA/EST data available from plant transcriptome sequencing projects (Additional file 2). Only a few cases have been described for myosins from *Oryza sativa*[35] and *Arabidopsis thaliana*[30] that report intron retention events and alternative transcription start sites. We did not find any alternative splicing event in the available cDNA/EST data and the reported intron retention cases are not even conserved in closely related species leading to completely different sequences, frame-shifts and in-frame stop codons. Therefore, we conclude that either the reported cases contain incompletely spliced transcripts or that alternative splicing in plants is species-specific in contrast to the strong inter-species conservation of the coding sequence.

### Phylogenetic analysis, classification and nomenclature

All new plant myosin sequences have been added to a multiple sequence alignment including all annotated myosins of all classes [23]. This is a structure-guided sequence alignment in which gaps are prohibited within sequence regions mapping to secondary structural elements of the crystal structure of the myosin motor domain. Wherever gaps were present in genome assemblies leading to missing exons, we kept the integrity of the coding sequence of the neighbouring exons. Myosins are usually classified based on phylogenetic analyses of their motor domain sequences [23,36]. While it is agreed that new classes are defined by strongly supported phylogenetic groupings and conserved domain organisations, a concise nomenclature of multiple members within these classes has not been developed yet. Such a nomenclature should reflect the phylogenetic relation of different subtypes within classes and thus needs to comply with branch- and species-specific whole genome, genomic region and single gene duplications leading to orthologs and paralogs.

**Figure 1:**
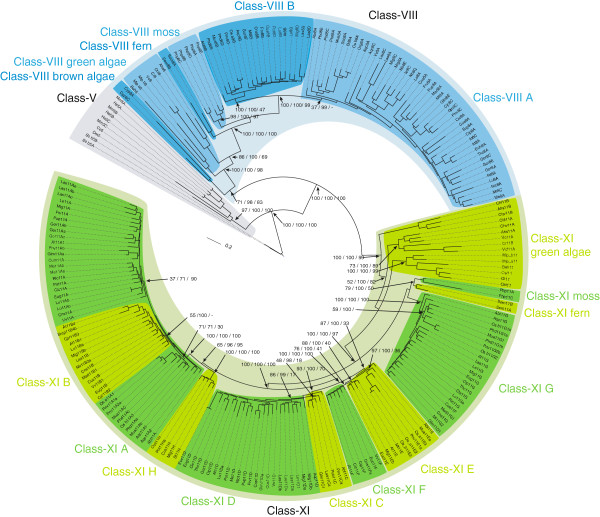
**The phylogenetic tree of selected plant myosins reveals distinct subtypes.** Maximum-likelihood topology generated under the JTT + Γ model in RAxML showing branch lengths for the motor domains of 221 ingroup class VIII and XI myosins and five class V outgroup myosins. CD-Hit (90% idenity) was used to obtain a representative dataset for subtype classification and visualization. Support for the major branchings indicating the grouping of the plant myosins into different class VIII and class XI subtypes is given as posterior probability (MrBayes), likelihood bootstrap (RAxML) and neighbour-joining bootstrap (ClustalW), all in percentages (the trees including all branch support values are available as Additional file 3). Despite the general strong support for major branches by all methods (like the separation of spermatophytes from mosses and ferns), some of the subtype groupings are not similarly supported. For instance, the separation of subtype 11C from subtype 11D myosins, which is well supported by MrBayes (posterior probability support of 98%), is only poorly supported in the neighbour-joining tree (18%). Class V myosins of *Caenorhabditis elegans, Drosophila melanogaster, Homo sapiens, Mus musculus* and *Saccharomyces cerevisiae* were used as outgroup. The scale bar corresponds to estimated amino acid substitutions per site. All species abbreviations used in the tree are listed in Figure 2 and Additional file 13.

**Figure 2:**
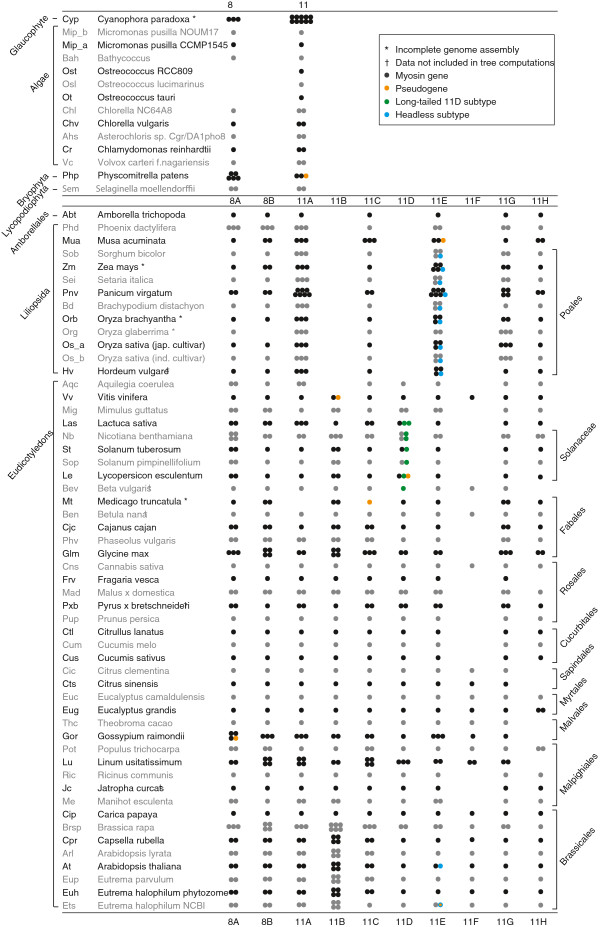
**Inventory of myosin subtypes in plant species with available genome assemblies.** The myosins of the Glaucophyte *Cyanophora paradoxa* and the 67 completely sequenced plant species were ordered according to subtypes. Each dot represents a myosin gene. Homologs of myosin-8A and -8B, named -8C, -8E, -8G, …, and -8D, -8F, -8H,…, respectively, were grouped together in the columns for myosin-8A and -8B. The species are sorted according to major branches for better orientation and comparison. Species abbreviations as used within this study are given in front of the species names.

The analysis of the assembled plant myosins showed that, as has been found previously, the plants (green and red algae, land plants) encode myosins of two major subfamilies, the class VIII and class XI myosins [23], which further split into several subtypes. The phylogenetic trees of the plant myosin motor domains revealed the same subtypes independently of which method had been used for tree reconstruction (Figure 1, Additional files 3 and 4). Accordingly, we suggest the following nomenclature that reflects the many whole genome duplications, which happened during spermatophyte evolution:

Class VIII myosins: The spermatophyte class VIII myosins group into two major subtypes that we named A and B in accordance with others [30]. These two subtypes are the result of an ancient single gene or genome duplication in the common ancestor of all extant angiosperms. Due to additional branch-specific duplications many plants encode more than two class VIII myosins. Because these additional homologs do not correlate across branches and extant species contain different sets of subtypes we named subtype A homologs A,C,E,G,… and subtype B homologs B,D,F,H,… This way, the membership to one of the two major subtypes becomes apparent. For example, *Solanum tuberosum* contains the myosins-8A, -8B and -8C, while *Medicago truncatula* encodes myosin-8A, -8B, and -8D (Figure 2). The subtype classification based on the phylogenetic tree is in agreement with the gene structures. The class VIII subtype A and subtype B myosins of the spermatophytes have identical gene structure patterns (intron positions at exactly the same positions; Additional file 5). The only intron, that does not align, is the intron located in the first unique region after the IQ motifs in the C-terminal tail domain. In subtype B myosins, the remainder of the tail is encoded within a single exon. In subtype A myosins, the tail is interrupted by a conserved intron located at the C-terminal end of the conserved C-terminal region 1 (see below). This intron position can also be taken for discriminating A and B subtypes.

Class XI myosins: Similar to the naming scheme for class VIII myosins, spermatophyte class XI myosins were named A to H according to their branching in five major subgroups. Out of these, three subgroups were further refined into subtypes 11A/11B, 11C/11D and 11E/11F. Additional numbers and characters reflect further, branch specific duplications. Numbers mark duplications affecting whole branches, while homologs in single species, which underwent additional duplications, are described by lowercase letters. For example, *Brassica rapa* underwent a species-specific whole genome duplication in addition to a whole genome duplication at the origin of the Brassicales clade [37]. Accordingly myosin homologs of subtype B encoded by *Brassica rapa* are named myosin-11B1, -11B2a, -11B2b, -11B3, -11B4a, and -11B4b. The ortholog (numbers) and paralog (lowercase letters) relationship becomes apparent immediately. In contrast to the class VIII myosins, the class XI myosins have completely conserved gene structures. Some myosins have lost single introns in the tail regions but these losses are not subtype specific and cannot be used as discriminator.

Altogether, 208 of the plant myosins grouped to class VIII and 594 to class XI. 187 of the class VIII and 565 of the class XI myosins were derived from whole genome sequencing projects of 67 plant species (Figure 2).

### Class VIII myosins

**Figure 3:**
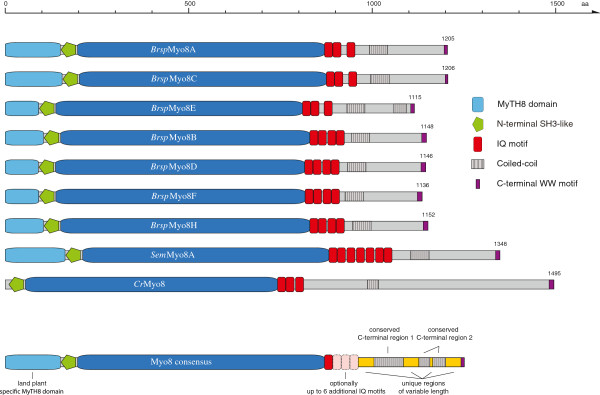
**Domain organisation of class VIII myosins.** The myosin motor domain, an N-terminal SH3-like domain, 1 to 7 IQ motifs, a coiled-coil region separated by unique parts and a C-terminal motif defined by two consecutive tryptophans (WW-motif) characterize myosins of class VIII. Tracheophyta also have an N-terminal region in common that we suggest naming the MyTH8 domain. Examples for domain organisations are given by the class VIII myosin sequences of *Brassica rapa* (*Brsp*). In general, homologs of the A-branch (myosin-8A, -8C, -8E) contain 3 IQ motifs, while homologs of the B-branch (myosin-8B, -8D, -8F, -8H) have 4 IQ motifs. In addition, the domain organisations of myosin sequences from *Selaginella moellendorffii* (*Sem*) and *Chlamydomonas reinhardtii* (*Cr*) are shown. They illustrate examples for domain architectures with 7 IQ motifs (*Sem*Myo8A) and without the N-terminal MyTH8 domain (*Cr*Myo8). The class VIII consensus domain architecture is depicted by the lowermost scheme entitled “Myo8 consensus”. All domain schemes are drawn to scale. Small numbers at the C-termini denote the number of residues of each sequence.

Class VIII myosins were found in all viridiplantae except the *Ostreococcus* green algae. They consist of an N-terminal SH3-like domain (Additional file 6), a motor domain without any class-specific extended loops, three to four IQ motifs for binding calmodulin and calmodulin-like proteins, and a C-terminal tail including coiled-coil regions separated by unique regions (Figure 3). At the C-termini they end with a characteristic motif containing two consecutive tryptophans (Additional file 7). The land plants also contain an N-terminal extension characterised by several conserved motifs (Additional file 7) that we suggest to name MyTH8 domain in accordance with other domains first observed and described in myosins (MyTH1 and MyTH4 domains). However, not all motifs are included in each MyTH8 domain. Because the entire MyTH8 extensions are encoded by single exons they were unambiguously identified although the overall similarity is quite low. We suggest naming all these extensions MyTH8 domains, in order to avoid introducing sub-categories with different names for the extensions depending on motif compositions. Outliers to this general domain architecture are the *Selaginella moellendorfii* (*Sem*) class VIII myosins, which contain seven IQ motifs, and a subclass of the myosins of the Rosaceae, which encode only one IQ motif, in contrast to the three and four IQ motifs found in all other class VIII myosins (Figure 3). In the phylogenetic tree of the class VIII myosins, the algae homologs form a group separating at the origin of the class followed by the fern (*Selaginella moellendorfii*; *Sem*) and moss (*Physcomitrella patens*; *Php*) myosins. The various *Sem* and *Php* myosins are the result of sub-branch or species-specific duplications. The tracheophyte class VIII myosins group into two distinct groups “A” and “B”. Further duplicates are part of one of these groups and, therefore, the result of gene or genome duplications within sub-branches of the tracheophytes (Figure 2). In general, type “A” class VIII myosins contain all motifs of the MyTH8 domain but miss the third IQ motif, while type “B” homologs miss some of the MyTH8 domain motifs but contain four IQ motifs. Exceptions are monocotyledon type “A” myosins that also comprise four IQ motifs, and several single examples showing sequence- and species-specific deviations of the general domain composition.

### Class XI myosins

**Figure 4:**
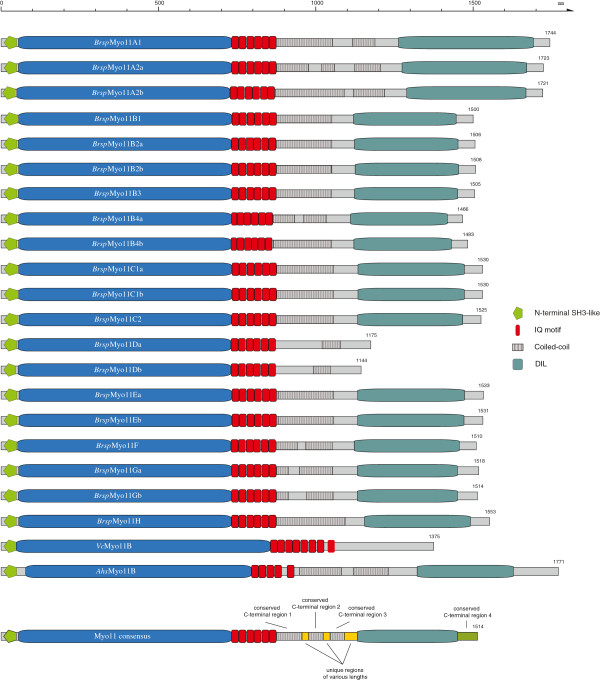
**Domain organisation of class XI myosins.** Land plant class XI myosins are composed of an N-terminal SH3-like domain, the myosin head domain, 6 IQ motifs, a coiled-coil region with unique parts of variable length and a C-terminal DIL domain. The C-terminal DIL domain was lost in most myosins of subtype 11D and in some algae class XI myosins. The domain organisations of the different subtypes are illustrated by *Brassica rapa* (*Brsp*) myosins. Myosins of the green algae, e.g. *Volvox carteri f. nagariensis* (*Vc*) and *Asterochloris sp. Cgr/DA1pho* (*Ahs*), show different domain compositions with respect to the number of IQ motifs. *Vc*Myo11B has also lost the C-terminal DIL domain like the myosin-11D homologs. A consensus domain composition is depicted in the lowermost scheme (Myo11 consensus). All domain schemes are drawn to scale. Numbers at the C-termini denote the lengths of the respective sequences.

**Figure 5:**
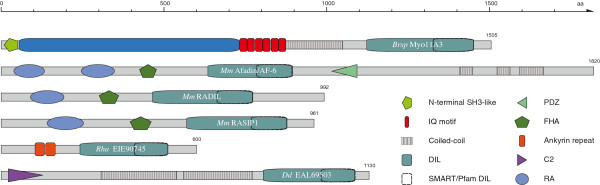
**Domain organisations of DIL domain containing proteins.** Here, the domain architectures of DIL domain containing proteins are shown. DIL domains are found in myosins of class V and XI, in Afadin/AF-6, RADIL (Ras association and DIL domains), RASIP1 (Ras interacting protein 1) and in uncharacterized fungal and amoebae genes. As examples, domain organisations are shown for *Brassica rapa* (*Brsp*) myosin-11B3, *Mus musculus* (*Mm*) Afadin/AF-6, *Mm*RADIL, *Mm*RASIP1, EIE90745 (GenBankID) from *Rhizopus arrhizus RA 99–880* (*Rha*), and EAL69503 from *Dictyostelium discoideum AX4* (*Dd*). Domain schemes are drawn to scale. Small numbers at the C-termini denote the lengths of the respective protein sequences.

In general, class XI myosins consist of an N-terminal SH3-like domain (Additional file 6), the motor domain, several IQ motifs for binding calmodulin, coiled-coil regions and a C-terminal tail containing a DIL domain (Figure 4, [23]). In current domain prediction databases like SMART [38] and Pfam [39], the DIL domain is restricted to about the C-terminal third of the original description [40]. Because in class XI myosins the tail sequences C-terminal to the coiled-coil regions are highly conserved suggesting a common domain we reassessed the definition of the DIL domain. Based on TBLASTN and PSI-BLAST searches DIL domains were found in class V and class XI myosins, Afadin/AF-6, RADIL (Ras association and DIL domains), RASIP1 (Ras interacting protein 1), and in uncharacterized fungal/yeast and amoebae genes in combination with ankyrin repeats and C2 domains, respectively (Figure 5). The multiple sequence alignment showed, that the conserved part of all these sequences comprises about the DIL domain as it has originally been described [40]. This extended region also represents the part of the tails of the two myosin V homologs of *Saccharomyces cerevisiae* that have been found to be protease stable [41]. The structures of these yeast myosin V tail regions show two subdomains that are interconnected by a long α-helix [41,42]. Based on the crystal structures and the sequence alignment of the DIL domains we here adapt the original description of the DIL domain and suggest updating the domain databases accordingly.

**Figure 6:**
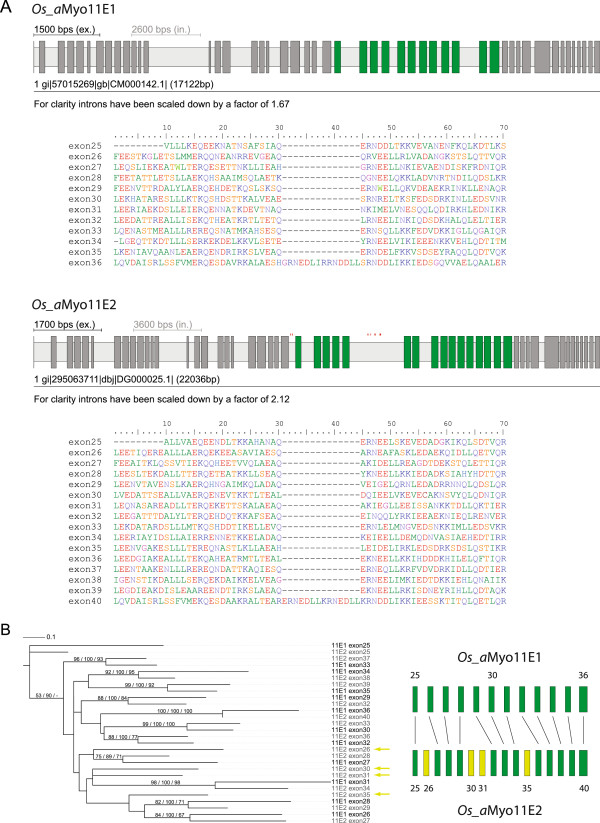
**Exons encoding the coiled-coiled regions of Liliopsida myosin-11E1 and myosin-11E2. A)** The Liliopsida subtype 11E1 and 11E2 myosins contain clusters of exons with identical lengths, reading frame, splice sites, and sequence similarity similar to mutually exclusive spliced exons. However, cDNA clones are available spanning the first few exons of the clusters indicating that all exons of the cluster are most probably constitutively spliced. Here, the gene structures of *Oryza sativa japonica group* (*Os_a*) myosin-11E1 and myosin-11E2 are shown as examples. The clusters of similar exons span exons 25–36 of myosin-11E1 and exons 25–40 of myosin-11E2. Exons are denoted as dark-grey and introns as light-grey bars. The similar exons of the clusters are coloured in green. Sequence similarity of the corresponding exons is shown in the multiple sequence alignments below the respective gene structures. **B)** The phylogenetic tree of exons 25–36 of the 11E1 variant and exons 25–40 of the 11E2 variant shows that the exons of both variants correspond to each other with the exception of exons 26, 30, 31 and 35 of variant 11E2. Phylogenetic trees were calculated with RAxML (shown), MrBayes and ClustalW, and bootstrap values (in percentages) and posterior probabilities for the nodes are given.

The phylogenetic tree of the plant myosins revealed eight different and distinct subtypes (Figure 1). All class XI myosins except the variant 11A, the short variant 11D, and Liliopsida variant 11E myosins contain six IQ motifs, three highly conserved coiled-coil regions interrupted by short unique regions, the DIL domains and a class XI specific C-terminal tail motif (Figure 4). The C-termini of the tails of the yeast class V myosins, although not part of the DIL domain definition, form α-helices that fold back to the N-termini of the DIL domains via long unstructured loops [41,42]. However, the sequences comprising these α-helices are not similar between the two yeast class V myosins, and they do not show any similarity to the C-termini of the class XI myosins. The C-termini of the class XI myosins are conserved between class members (Additional file 8) and it is anticipated that they form α-helices like the class V myosins. The short-tailed variant 11D myosins miss the DIL domain, and some of them have unique regions instead of the conserved C-terminal region 1 and 2 (see section below). Some of the variant 11A myosins contain long insertions before and/or after the conserved C-terminal region 2. Variant 11E1 and 11E2 type myosins have very long coiled-coil coding regions instead of the conserved C-terminal region 2 (Figure 6A). The insertions consist of ten and fourteen exons, respectively, that have identical reading frames, identical split codons at 5′ and 3′ exon borders, and similar sequences (Figure 6A). Based on their splice site patterns these exons could be incorporated in a mutually exclusive manner. However, two cDNA clones from *Festuca arundinacea* [GenBank:GO853568, GenBank:DT691477] cover exons 21 to 27 of the type 11E2 myosins supporting that all exons of the insertion are constitutively spliced. The exons of the myosin-11E1 and -11E2 insertion correspond to each other except for exons 26, 30, 31, and 35 of myosin-11E2 that have either been gained in the 11E2 variant or lost in the 11E1 variant (Figure 6B). The phylogenetic tree of the exon sequences does not support ancestry of the additional exons in the 11E2 variant through duplication from neighbouring exons, and therefore their loss in the 11E1 variant is more likely.

### Plant headless myosins

**Figure 7:**
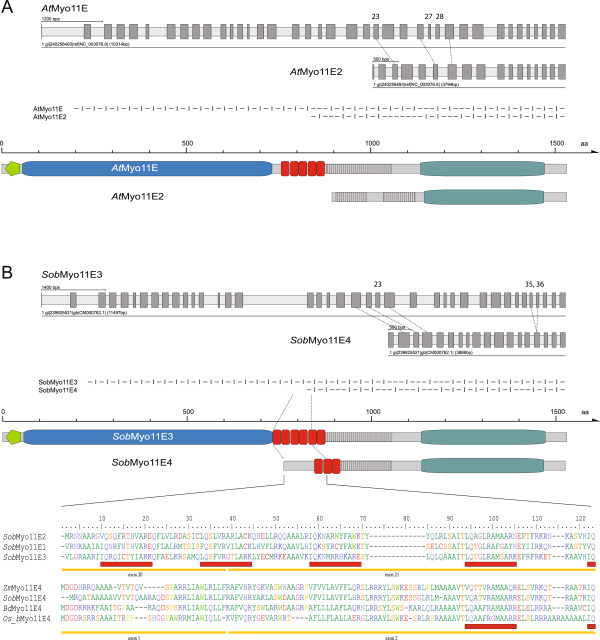
**Headless class XI myosins. A)** Headless myosins of subtype myosin-11E2 were identified in *Arabidopsis thaliana* and *Eutrema halophilum* (NCBI assembly). These myosins are subtype myosin-11E duplicates, which have lost the myosin head domains or were derived by partial duplication of the myosin-11E tail domains. Gene structures and domain architectures of *Arabidopsis thaliana* (*At*) myosin-11E and myosin-11E2 are shown for comparison. Exons 1 to 22 and exons 27 and 28 (encoding the unique region) of *At*Myo11E have been lost in *At*Myo11E2, resulting in the loss of all domains but the coiled-coil and DIL domains. Below the gene structure schemes, an alignment of the gene structures is given, in which exonic sequences are represented by hyphens ("-") and introns by vertical bars ("|"). Most introns of both sequences have the same position and phase demonstrating their common ancestry. **B)** Headless myosins of subtype 11E4 are found in all members of the Poales clade. For instance, *Sorghum bicolor* (*Sob*) myosin-11E4 lost exons 1 to 20 and exon 23 compared to *Sob*Myo11E3. Therefore, subtype 11E4 myosins encode only 3 IQ motifs and miss part of the coiled-coil region. Gene structure conservation and loss of the intron between exons 35 and 36 (11E3 numbering) in myosin-11E4 is shown in the exon-intron pattern below the gene structures. Differences in the domain architectures of subtype 11E3 and headless 11E4 myosins are highlighted in the domain organisation scheme and the multiple sequence alignment. In this alignment, residues encoded by exons 20 and 21 of *Sob*Myo11E1, *Sob*Myo11E2, and *Sob*Myo11E3 were aligned with those encoded by exons 1 and 2 of headless myosin-11E4 from *Zea mays B73* (*Zm*Myo11E4), *Sorghum bicolor* (*Sob*Myo11E4), *Brachyopodium distachyon* (*Bd*Myo11E4) and *Oryza sativa indica group* (*Os_b*Myo11E4). Below the sequences, yellow lines and red bars indicate exons and IQ motifs, respectively.

Two types of headless plant myosins have been found that are the results of (potentially partial) duplications of myosins of subtypes 11E and 11E3 (Figure 7). The subtype 11E duplication has been observed in *Arabidopsis thaliana* (*At*) and *Eutrema halophilum* (*Ets*; NCBI assembly), but not in *Arabidopsis lyrata*, the phytozome *Eutrema halophilum* assembly, *Eutrema parvulum* or any other species. In *A.thaliana*, the headless myosin-11E2 is transcribed and expressed but does not show any discernible phenotype [30]. This *At*Myo11E2 is arranged in tandem to the Myo11E myosin and thus most probably the result of a recent single gene duplication (Additional file 9). *At*Myo11E2 is not only headless but does also not contain the neck region (no IQ motifs) and the unique region encoded by exons 27 and 28 (*At*Myo11E numbering; see Figure 7A). After duplication of the *At*Myo11E gene, the tail-coding exons 23 to 39 have been retained in *At*Myo11E2 and two additional exons have been added to the 5′ end extending the coiled-coil region encoded by exons 23 to 26 (*At*Myo11E; corresponds to exon 3 to 6 in *At*Myo11E2; Figure 7A). Myosin-11E2 in *E.halophilum* (NCBI assembly) is a similar duplication of the *Ets*Myo11E gene that, however, has additionally lost exon 23, 25 and 26, and starts somewhere within exon 24 (Figure 7A). Because the 5′ end is not defined and EST data are not available, the *Ets*Myo11E is most probably a pseudogene. Since similar headless copies of Myo11E have been found in two species of the Brassicales clade it seems likely that the gene duplication event and immediate subsequent loss of the exons encoding the motor domain and IQ motifs happened at the origin of the Brassicales. The loss of the remainder of the tail could be a slower process and not completely finished yet in *E.halophilum*. Alternatively, *E.halophilum* and *A.thaliana* might have duplicated the gene independently of each other. In *A.thaliana*, the tail region has been converted to a new functional gene after separation from *A.lyrata* that is probably still in the process of sub- or neo-functionalization.

In contrast, myosin-11E3 duplicates (called Myo11E4) have been found in all sequenced species of the Poales clade and are supported by EST/cDNA data for several of the species. These subtype 11E4 myosins encode three IQ motifs, miss the first part of the coiled-coil region of the Myo11E3 homologs due to loss of exon 23, but contain a conserved 40 amino acid long N-terminal extension (Figure 7B). They are not as identical to their respective Myo11E3 homologs as *At*Myo11E2 is to *At*Myo11E (48% identity compared to 72% for the *A.thaliana* homologs) and they are independently located in the genome and not in tandem to the Myo11E3 homologs (Additional file 9). This suggests that sub- or neo-functionalization has already occurred.

### Short-tailed class XI myosins

**Figure 8:**
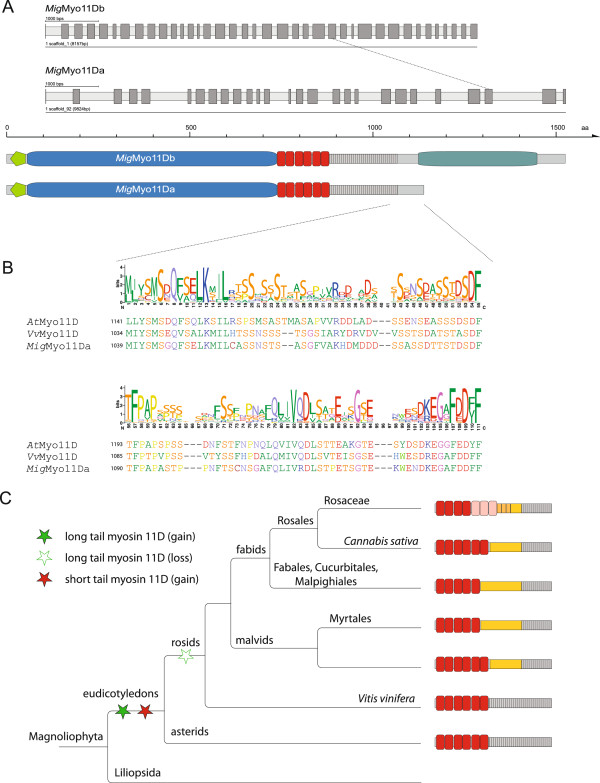
**Eudicotyledon subtype 11D myosins. A)** Plants of the asterids clade encode long-tailed 11D myosins containing a C-terminal DIL domain in addition to the typical, short tailed 11D myosins lacking the DIL domain. As examples for 11D myosins, the *Mimulus guttatus* (*Mig*) short tailed 11Da and long tailed 11Db myosins are shown. All but the last two exons of *Mig*Myo11Da are in accordance with corresponding exons of the long tailed version *Mig*Myo11Db. **B)** The C-terminus of the short tailed myosins (encoded by the last two exons) is conserved throughout eudicotyledons. The level of conservation is displayed by the WebLogo plot [43]. As examples, C-terminal protein sequences of the asterid myosin *Mig*Myo11Da and rosid 11D myosins from *Arabidopsis thaliana* (*At*) and *Vitis vinifera* (*Vv*) are shown. The numbers in front of each sequence in the alignment correspond to sequence positions. **C)** The cladogram shows the taxonomy of Magnoliophyta subtaxa and species. After separation of the eudicotyledons clade from the Liliopsida, long tailed 11D myosins and, subsequently, short tailed duplicates were invented. Most probably, the long tailed 11D myosin was lost in the last common ancestor of the rosids branch.

The subtype 11D is specific to species of the eudicotyledon branch and must have therefore been invented after separation from the Liliopsida (Figure 8). In general, myosins of this subtype are short-tailed. They miss all domains C-terminal to the coiled-coil regions of the normal class XI myosins but instead have a class-specific conserved C-terminal domain (Figure 8B, Additional file 8). Interestingly, the species of the asterids clade encode subtype members that still have the long tails, identical in domain organisation and gene structure to the other class XI myosins (Figure 8C). The long myosin-11D tails group to their short-tailed homologs in the phylogenetic tree of the myosin-11 tails (Additional file 3). Thus, it is very unlikely that such a long-tailed subtype 11D myosin would have been built by the fusion of a short-tailed myosin-11D with a copy of a tail of one of the other class XI myosins. Rather, the ancestor of the eudicotyledons contained a long-tailed myosin-11D and the short-tailed myosin-11D appeared as result of a gene or whole genome duplication (Figure 8C). The myosin-11D invention happened at about the time of the γ pan-eudicotyledon triplication, and the subsequent duplication resulting in the short-tailed subtype 11D myosins could thus also be part of this triplication. Subsequently, the long-tailed version has only been retained in the asterids branch and in *Beta vulgaris*. The short-tailed and long-tailed class 11D myosins have identical gene structures up to exon 25 (Figure 8A). Their unique 100 amino acids long tail is encoded by two exons.

## Discussion

Evidence for WGDs can be found by various methods. One of these is the reconstruction of phylogenetic trees from DNA and protein sequences. When analysing gene and protein families in phylogenetic analyses, however, it is very difficult to distinguish between single gene duplications, the duplication of small genomic regions, and WGDs. Theoretically, WGDs lead to the doubling of the entire gene set. However, species cannot maintain the entire set of duplicates because this provides the basis for deleterious mutations that would compromise the fitness of the genome [44]. Therefore, duplicated genomes transform back to the original state by eliminating most of the duplicated gene set. Duplications of genomic regions can be distinguished from single gene duplications due to the micro-syntheny that should be present in the first case. In contrast, single gene duplications often result in tandemly arrayed genes. The difficulties in distinguishing between the three types of gene and genome duplications can be overcome through the analysis of multiple independent genes. If multiple genes from different genomic regions were independently duplicated in one genome compared to another, this would strongly support a WGD. Here, we propose using the myosin motor protein family as marker for WGDs in plants. Plant myosins represent a multi-gene family whose members are independent and distributed over all chromosomes in *Arabidopsis thaliana* (example of an eudicot) and *Oryza sativa* (example of an monocot; Additional file 9). In addition, we use a very high taxonomic sampling. This allows for the direct comparison of species and branches, which have undergone recent WGDs, to many closely related species/branches that did not duplicate. The first step of our analysis therefore consisted in the identification of the myosin repertoire in as many species as possible.

The complete repertoire of all myosins within a species can only be determined by analysing its genome sequence. Transcriptome data like cDNA, EST and RNASeq data are never complete because not all developmental stages and cell types are covered, and because not all myosins are abundant. By analysing transcriptome data it can therefore never be decided whether a certain myosin subtype is really “absent” in this species or only absent in the data. Another drawback of transcriptome data are usually their short read length. Given the above-average length of the myosin motor domain (compared to the average protein length in eukaryotes) cDNA and EST reads would be spread over the entire motor domain sequence. At the normal read depth of transcriptome data it would thus not be possible to decide which N-terminal read would belong to which read mapping to the middle or C-terminus of the motor domain, or whether these would belong to gene duplicates. The unknown number of gene duplicates in the species to be analysed is a further limitation. Short, non-overlapping sequences can, however, not be used in phylogenetic tree reconstructions. Therefore, we only used data from whole genome and high coverage assemblies. Incomplete genome assemblies as result from low coverage sequencing were not included into the analysis. Examples for the latter are the fragmentary assemblies of *Penstemon cyananthus*, *Amaranthus tuberculatus*, *Lotus japonicus*, *Vigna radiata* and *Leersia perrieri*. Unfortunately, a genome sequence of a gymnosperm is not available today. Therefore, whole genome duplications in plants can only be traced back to the last common ancestor of the angiosperms.

Annotated gene datasets are only available for a few sequenced plant genomes, and most of these annotations are based on automatic gene predictions without including cDNA and EST data. Full-length cDNA sequences are only available for the *Arabidopsis thaliana* and *Oryza sativa* sequencing projects covering a few of the myosins. Therefore, we had to manually assemble all sequences based on preliminary results from *ab-initio* gene prediction and cross-species gene reconstruction software. To help in the correct assembly of the myosin coding sequences from the genomic DNA, available cDNA sequences of single homologs from other species have also been used for comparison and are included in the multiple sequence alignment. Altogether, we were able to identify and reconstruct 775 myosins in 67 completely sequenced plant species (Figure 2). In the qualitative analysis of the presence and absence of homologs in species and branches we included all sequences while only complete and “partial” (see Results section for definition) sequences were used in the tree computations. These phylogenetic trees were used to resolve the ortholog-paralog relationship between the analysed plant myosins. The grouping into different myosin subtypes is additionally supported by subtype-specific identical gene structures (Additional file 5) and subtype-specific homologous sequences within the unique regions of the class VIII and class XI myosins (Additional file 10). By mapping the paralogs onto the plant species tree, it can subsequently be determined whether the paralogs resulted from a duplication event before or after a given branching event. In the case of a WGD we suppose that many if not all of the myosins are present as duplicates. It is highly unlikely that several myosin subtypes duplicated independently of each other, e.g. as part of multiple single gene duplications. In contrast, if only one or two of the myosins were duplicated in the comparison of two closely related species/branches, it would be rather likely that these duplications are the result of single gene duplications or duplications of genomic regions.

**Figure 9:**
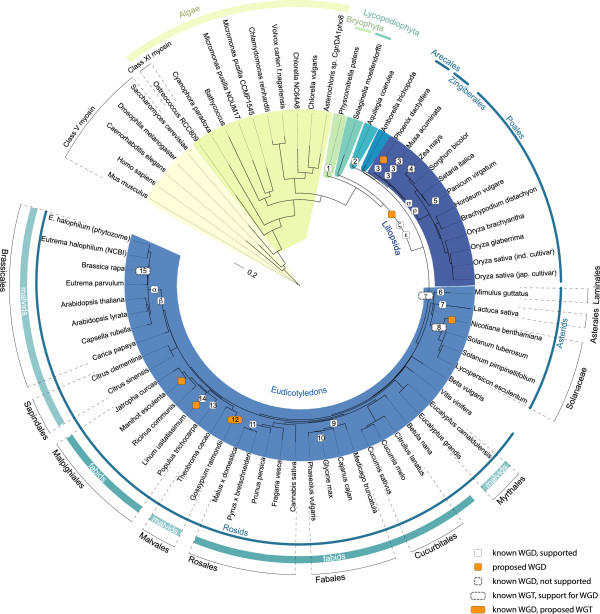
**Whole genome duplications in land plants.** Maximum-likelihood topology generated under the JTT + Γ model in RAxML showing branch lengths for 65 class VIIIA myosin motor domains. The class XI myosin of *Ostreococcus* and 5 metazoan class V myosins were included as outgroup. The tree was used as reference species tree to map formerly described and newly proposed WGDs and genome triplication events (WGTs) on the respective branches. Branches are labelled for better orientation. White boxes indicate WGDs described in the literature. The numbers point to the first publications describing the respective event. Letters are shown for duplications that are usually referred to by Greek letters. Orange boxes indicate newly proposed WGDs. The numbers in boxes refer to the following publications: 1 (*Physcomitrella*) - [46], 2 (*Ranunculales*) - [47], 3 (*Zingiberales & Arecales*; the WGDs in the *Musa acuminata* branch are also called α, β, and γ) - [3], 4 (*Zea*) - [48], 5 (*Panicum*) - [49], 6 (*Phrymaceae*) - [50], 7 (*Lactuca*) - [6], 8 (*Solanum*) - [51], 9 (*Phaseoleae*) - [52], 10 (*Glycine*) - [53], 11 (*Malus*) - [8], 12 (*Gossypium*) - [4], 13 (*Populus;* also called ρ) - [54], 14 (*Linum*) - [55], 15 (*Brassica*) - [37], α, β (*Brassicaceae*) and γ (core eudicotyledons) - [56], ϵ (angiosperms) and ζ (spermatophyta) - [14], ρ and σ (*Poaceae*) - [57]. The topology of the tree is in general accordance with those obtained by ClustalW (Neighbour joining) and MrBayes (Additional file 11). The scale bar corresponds to estimated amino acid substitutions per site.

In order to derive a species tree of the analysed 67 plants we computed a phylogenetic tree of the myosin-8A subtypes, which are present in all species except the *Ostreococcus* algae (Figure 9, Additional file 11). The derived phylogeny, however, is in a few cases inconsistent with the most widely accepted species phylogeny. These inconsistencies include the placing of *Aquilegia coerulea*, *Amborella trichopoda*, and malvid and fabid sub-branches. Both *Aquilegia* and *Amborella* myosin-8A types group to the Liliopsidan branch instead of being sister to core eudicotyledons and angiosperms, respectively. Also, the Myrtales (*Eucalyptus* species) and the Malvales (*Gossypium raimondii* and *Theobroma cacao*) do not group to the other malvids of the Sapindales and Brassicales branches. While Myrtales often group basal to fabids and malvids [18,45], the Malvales are usually always found together with the other malvids. However, the suitability of this myosin-8A tree to map WGDs happened during angiosperm evolution remains unaffected by these discrepancies.

**Figure 10:**
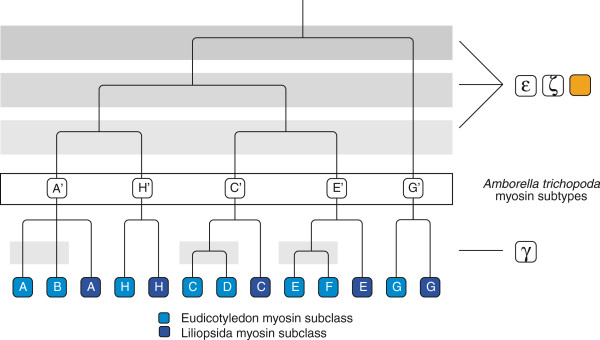
**Supposed emergence and evolution of class XI subtypes by ancient whole genome duplications.** The basal angiosperm *Amborella trichopoda* (*Abt*), which diverged before the split of the monocot and the eudicot lineages, encodes a myosin homolog of each of the major five subtypes A’, C’, E’, G’ and H’. The emergence of these myosin-11 subtypes can be explained by three WGDs, of which two are potentially identical with the ϵ and ζ WGD. Alternatively, two of the myosin-11 subtype duplications could be the result of single gene duplications. Subtypes B, D and F, which are present in eudicots but not in monocots, are probably the result of the γ hexaploidy event.

A key to mapping the plant WGDs is the analysis of the *Amborella trichopoda* genome. *Amborella* is sister to the ancestor of the monocots and eudicots, and diverged after the two ancient WGDs ϵ and ζ happened in angiosperm evolution [14]. Thus, it should contain traces of these WGDs but not from the γ hexaploidy event at the origin of the core eudicots and from the ρ and σ WGDs at the origin of the monocots. The *Amborella* genome contains five class XI myosins belonging to five subtypes that appear outside the monocot and eudicot myosins in the phylogenetic trees (Figure 10). These can therefore be regarded as ancient subtypes 11A’, 11C’, 11E’, 11G’ and 11H’, grouping as (((A’H’),(C’E’)),G’). These five myosin-11 subtypes could be a remnant of the two angiosperm wide WGDs ϵ and ζ [14], which were dated back to the diversification of extant angiosperms (ϵ, 192 Mya) and the diversification of extant seed plants (ζ, 319 Mya), respectively (Figure 10). However, because a gymnosperm genome is currently not available, the myosin data did not reveal the exact timing of these duplications. It will be highly interesting to see, which myosin-11 types or supergroups (e.g. common ancestor of A’H’ or (A’H’),(C’E’)) gymnosperms contain. All monocots only contain class XI myosins of subtypes 11A, 11C, 11E, 11G and 11H (Figures 2 and 10) while eudicots also contain myosins of subtype 11B, 11D and 11F. These eudicot-specific subtypes could be the result of the hexaploidy event at the origin of the core eudicots (Figure 10). We suppose that the ancient monocot and eudicot genomes contained one of each of the respective myosin subtypes. Many extant eudicots still contain just one of each of the myosin subtypes. The subtypes 11F and 11H have been lost in some branches and single species. A few species encode single gene duplicates compared to their closest relative that have obviously been derived by single gene duplications, like the head-less myosin-11E subtypes in *Arabidopsis thaliana* and *Eutrema halophilum* (NCBI assembly) and the myosin-11H duplication in *Eucalyptus grandis*. While the class XI myosins show support for WGDs back to the last common ancestor of the seed plants, the class VIII myosins only provide support for the most recent WGDs that happened after the γ-hexaploidy event and after the ρ- and σ-WGDs at the origin of the monocots.

**Figure 11:**
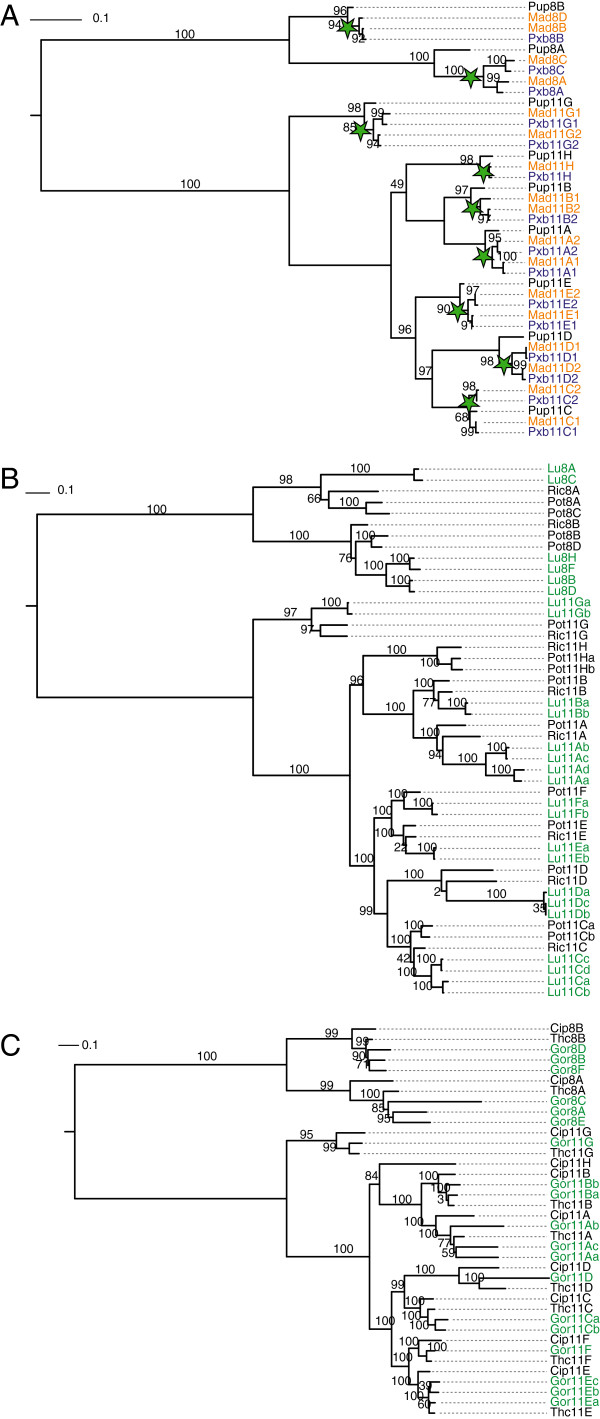
**Phylogenetic grouping of the myosin-11 subtypes suggests whole genome duplications. A)** Maximum-likelihood topology generated under the JTT + Γ model in RAxML showing branch lengths for all class XI myosin motor domains of the apple *Malus x domestica* (*Mad*), the pear *Pyrus x bretschneideri* (*Pxb*) and the peach *Prunus persica* (*Pup*). The WGD at the origin of the Maleae [8] is indicated by green stars. **B)** Maximum-likelihood topology generated under the JTT + Γ model in RAxML showing branch lengths for all class XI myosin motor domains of *Linum usitatissimum* (*Lu*), *Populus trichocarpa* (*Pot*) and *Ricinus communis* (*Ric*). **C)** Maximum-likelihood topology generated under the JTT + Γ model in RAxML showing branch lengths for all class XI myosin motor domains of *Gossypium raimondii* (*Gor*), *Theobroma cacao* (*Thc*) and *Carica papaya* (*Cip*). The topologies of the trees are in accordance with those of ClustalW and MrBayes (data not shown). The scale bars correspond to estimated amino acid substitutions per site.

Next, we looked for nodes at which considerable changes in the myosin repertoires have occurred. By analysing the myosin inventories and phylogenetic trees of myosin motor domains, we were able to both reconstruct formerly described ancient whole genome duplications and to propose additional ones (Figure 9). The myosin data support the α and β WGDs at the origin of the Brassicales (e.g. compare *Carica papaya*, which diverged before the WGDs, with any of the other Brassicales species) and the WGD at the origin of the legumes (Fabales, [9,52]). Instead of the triplication event at the origin of the Solanaceae [51] and the γ triplication preceding the rosid-asterid divergence, only genome duplications were identified. Also, the data support the ρ and σ WGDs at the origin of the Poales. In addition, many species specific duplications are supported like the WGD in *Brassica rapa*[5], the WGD in *Populus trichocarpa* (also called ρ, [54], Figure 11B), and the WGDs in *Glycine max*[9] and *Mimulus guttatus*[50] (Figure 9). The myosin data do not support the maize tetraploidy event [48], most probably because of the still fragmented maize genome assembly.

The qualitative analysis together with the phylogenetic tree reconstructions also allows for timing the WGD events. By resolving the phylogenetic relationship between species, we could, for example, support the proposed timing of WGDs in the Brassicales clade [15,56,58,59]. In accordance with these studies, the myosin data also support placing the α and β WGD events after the divergence of the Papaya lineage from the Brassicales clade (Figure 9). Similarly, the WGD found in *Malus x domestica*[8] was placed at the origin of the Maleae after their divergence from the Amygdaleae (containing *Prunus persica*, for instance; Figure 11A). We conclude that the myosin gene family could be very suitable for detecting ancient WGDs through phylogenetic reconstructions. Obviously, the plants retained many of the duplicated myosins after the WGD events and additional single gene duplications are rare. So far, the genes reported to have survived the ancient WGDs did mainly belong to transcription factors, transferases and their binding proteins, and protein kinases [14]. The most popular models to describe gene duplications include neo- and subfunctionalization, dosage effects, and shielding against deleterious mutations [60]. The reason for retaining so many similar myosins in plants has, however, not been determined yet. Myosins are not part of metabolic pathways, in which duplications of single genes have very strong effects, but are part of the intracellular transport machinery. Thus, duplicated myosins could have specialized in the transport of specific cargoes. Also, having a higher dosage of myosins after WGDs would probably not be harmful to the species.

In addition to the formerly described WGDs, we also found evidence indicating further WGDs (Figure 9, Additional files 3, 4 and 12): First, we found evidence for two very recent WGDs in *Linum usitatissimum*, of which only one had been suggested before [55]. The myosin-8B, myosin-11A and myosin-11C subtypes clearly group into one-to-two-to-four patterns and three of the 11D subtype myosins are still present (Figure 2, Figure 11B). It is unlikely, that seven independent myosins underwent single gene duplications or genomic region duplications in the short time since the divergence of *Linum* from *Ricinus* and *Populus*. Second, the myosin data indicated genome duplications in *Gossypium raimondii*. Recently, the genome of this cotton species had been sequenced independently by two groups [4,61]. One group analysed synonymous nucleotide substitution (*K*_S_) values and the resulting single peak had been interpreted as a single WGD [4]. The other determined an abrupt five- to sixfold ploidy in the cotton lineage shortly after its divergence from the ancestor shared with *Theobroma cacao* although *K*_S_ values also only showed a single peak [61]. In the genome analysis about 7,000 co-linearity supported gene triplets have been found [61]. The myosins are also present as triplets in subtypes myosin-8A, -8B, -11A, -11E and their phylogeny does not show any one-to-two-to-four pattern (Figure 2, Figure 11C). Thus, instead of two consecutive WGDs our data would support a triplication that happened after separation of *Theobroma* from *Gossypium*. For the exact timing genome data from additional species of the Malvales branch would be necessary. Third, the number of homologs encoded by *N.benthamiana* is doubled in comparison to other Solanaceae, with the exception of subtype myosin-11B, of which only three instead of four homologs were identified in *N.benthamiana*, (Figure 2, Additional file 12A). The *N.benthamiana* myosins always group together in single branches. Therefore, we propose a genome duplication in *Nicotiana benthamiana* after its divergence from the other Solanaceae. Forth, *Manihot esculenta* encodes duplicates of myosin-8A, -11A, -11B and -11E compared to *Jatropha curcas*, which encodes only one homolog of each of the myosin-11 subtypes (Figure 2, Additional file 12B). The one-to-two pattern of the duplicates indicates that the *Manihot esculenta* WGD happened after separation from *Jatropha* (Figure 9). Fifth, the myosin data suggest two WGDs or a genome triplication in the evolution of *Phoenix dactylifera* after its divergence from *Musa acuminata* (Figure 9). In detail, subtypes myosin-8A, -8B and -11A are present as triplets, subtypes -11E and -11G as duplets (Figure 2, Additional file 12C). In contrast, only a single WGD has been reported recently based on the analysis of a preliminary *P.dactylifera* annotation [3]. Sixth and most notably, reconstruction of the class XI myosin family suggests another duplication in the ancestor of angiosperms in addition to the ϵ and ζ WGDs (Figure 10). However, in this case we cannot distinguish between a single gene and whole genome duplication. This might become possible when genome assemblies of species become available that diverged after separation of the Lycopodiophyta but before the Magnoliophyta established.

In general, most whole genome assemblies were reported to contain only 80-90% genome coverage by comparing genome assembly sizes with experimental genome size estimations obtained by e.g. flow cytometry. Although most of the supposed missing genome sequence concerns telomere and other highly repetitive regions, myosin homologs might have been missed in our analysis due to gaps in the genome assemblies. However, the class VIII and class XI myosins consist of many subtypes. Even if one or several of the myosins were missing in a certain genome the comparison of the (incomplete) myosin repertoire of the genome to the presented table of myosins across the plant phylum (Figure 2) allows reconstruction of WGDs and will also allow prediction of WGDs in upcoming plant genome assemblies. The phylogenetic analysis of the myosins in these upcoming assemblies together with the dataset presented here will also allow the timing of proposed WGDs. This way, WGDs can already be reconstructed and predicted for species for which only fragmented genome assemblies are available hindering syntheny-based studies.

## Conclusions

Based on phylogenetic tree reconstructions, we identified two class VIII myosin subtypes and eight class XI subfamilies. The topology of the subtypes together with the phylogeny of the homologs within the subtype branches allowed reconstructing the WGDs that occurred in the evolution of the tracheophytes. Although most known WGDs could be reproduced the myosins did not reveal all known WGDs. Therefore, WGDs might have been missed in branches that do not show WGDs based on myosin data and for which further analyses are not yet available. The myosin data revealed evidence for two ancient, angiosperm-wide WGDs, potentially identical with the most ancient, formerly described WGDs occurring during seed plant and angiosperm evolution, called ϵ and ζ. In addition to reconstruct already known WGDs, we also propose further WGDs in the *Manihot esculenta*, *Linum usitatissimum*, *Gossypium raimondii*, *Nicothiana benthamiana* and *Phoenix dactylifera* lineages, and another possible WGD in the ancestor of the angiosperms. This is the first analysis of 67 completely sequenced plant genomes revealing most of the known WGD events by analysing a single protein family. We propose that myosin duplications not contained in the presented dataset but found in future sequenced species are very strong hints to further WGDs. The myosins will also be a strong complement where other methods are not appropriate of do not reveal clear answers.

## Methods

### Identification and annotation of the myosin heavy chain genes

The complete myosin heavy chain gene repertoires of *Chlamydomonas reinhardtii*, *Ostreococcus lucimarinus*, *Ostreococcus tauri*, *Populus trichocarpa*, *Arabidopsis thaliana*, *Sorghum bicolor*, and *Oryza sativa* were obtained from [23]. The sequences were updated based on newer genome assemblies if necessary. Some minor ambiguities in the tail regions were corrected based on the comparative analysis with newly available genomes from plants of the same branch. The myosin genes of most other plant and algae species have essentially been obtained as described in [23]. Shortly, myosin genes have been identified in TBLASTN searches starting with the protein sequences of the *Arabidopsis* myosins. The respective genomic regions were submitted to AUGUSTUS [32] to obtain gene predictions. However, feature sets are only available for a few plant species. Therefore, all hits were subsequently manually analysed at the genomic DNA level. When necessary, gene predictions were corrected by comparison with the other myosins as included in the multiple sequence alignment. As the amount of plant myosin sequences increased (especially the number of sequences from taxa with few representatives), many of the initially predicted sequences were reanalysed to correctly identify all exon borders in the unique parts of the tail regions. Where possible, EST data have been analysed to help in the annotation process.

Recently, genome sequencing efforts have been extended from sequencing species from new branches to sequencing closely related organisms. Within the plants these species include for example *Cucumis melo*, *Eucalyptus camaldulensis*, *Solanum pimpinellifolium*, *Lycopersicon esculentum*, *Eutrema halophilum* (two different assemblies of *Eutrema halophilum* (*Thellungiella halophila*) are available [62,63] that had been analysed independently here), and *Fragaria vesca*, of which the closely related species *Cucumis sativus*, *Eucalyptus grandis*, *Solanum tuberosum*, *Eutrema parvulum*, and *Prunus persica* had been sequenced before. Protein sequences from these closely related species have been obtained by using the cross-species functionality of WebScipio [34,64]. Nevertheless, for all these genomes TBLASTN searches have been performed. With this strategy, we sought to ensure that we would not miss more divergent myosin homologs, which might have been derived by species-specific inventions or duplications.

All sequence related data (protein names, corresponding species, GenBank ID’s, alternative names, corresponding publications, domain predictions, sequences, and gene structure reconstructions) and references to genome sequencing centres are available at CyMoBase (http://www.cymobase.org, [25]). A list of the analysed species, their abbreviations as used in the alignments and trees, as well as detailed information and acknowledgments of the respective sequencing centres are also available as Additional file 13. Most plant genomes have been published or are available from GenBank. Permission to use the myosin data from *Aquilegia coerulea*, *Citrus clementina*, *Eucalyptus grandis*, *Panicum virgatum*, *Phaseolus vulgaris* has been obtained from the genome project leaders. WebScipio [34,64] was used to reconstruct the gene structure (i.e. the exon/intron pattern) of each sequence.

### Generating the multiple sequence alignment

The plant myosin sequences were added to the structure-guided multiple sequence alignment obtained from [23]. In detail, we first aligned every newly predicted sequence to its supposed closest relative using ClustalW [65] and added it then to the multiple sequence alignment. During the subsequent sequence validation process, we manually adjusted the obtained alignment by removing wrongly predicted sequence regions and filling gaps. Still, in those sequences derived from low-coverage genomes many gaps remained. To maintain the integrity of exons preceded or followed by gaps, gaps reflecting missing parts of the genomes were added to the multiple sequence alignment. The sequence alignment can be obtained from CyMoBase or Additional file 10. Reduced alignments containing sets of representative sequences of less than 90% identity were obtained by using the CD-HIT suite [66].

### Computing and visualising phylogenetic trees

For calculating phylogenetic trees only complete and almost complete (missing a maximum of 5% of the supposed full-length sequence, “Partials”) sequences were included in the dataset (Additional file 1). As outgroup, class V myosin sequences from *Homo sapiens, Mus musculus, Caenorhabditis elegans, Drosophila melanogaster* and *Saccharomyces cerevisia*e were added. The phylogenetic trees were generated using three different methods: Neighbour Joining, Maximum likelihood and Bayesian inference. 1. ClustalW v.2.0.10 [65] was used to calculate unrooted trees with the Neighbour Joining method. For each dataset, bootstrapping with 1,000 replicates was performed. 2. Maximum likelihood (ML) analysis with estimated proportion of invariable sites and bootstrapping (1,000 replicates) were performed using RAxML [67]. To this end, ProtTest was used first to determine the most appropriate of the available 112 amino acid substitution models [68]. Within ProtTest, the tree topology was calculated with the BioNJ algorithm and both the branch lengths and the model of protein evolution were optimized simultaneously. The Akaike Information Criterion with a modification to control for small sample size (AICc, with alignment length representing sample size) identified the JTT model with gamma model of rate heterogeneity to be the best. 3. Posterior probabilities were generated using MrBayes v3.2.1. [69]. Using the mixed amino-acid option, two independent runs with 10,000,000 generations, four chains, and a random starting tree were performed. MrBayes used the JTT model [70] for all protein alignments. Trees were sampled every 1.000th generation and the first 25% of the trees were discarded as “burn-in” before generating a consensus tree. Phylogenetic trees were visualized with the CLC Sequence Viewer (http://www.clcbio.com) and iTOL [71] and are available as Additional files 3 and 11.

## Availability of supporting data

The data sets supporting the results of this article are included within the article (and its additional files). In addition, all data can be browsed at and obtained from CyMoBase (http://www.cymobase.org) [25].

## Competing interests

The authors declare that they have no competing interests.

## Authors’ contributions

MK assembled all sequences. SM performed data analyses. MK and SM wrote the manuscript. Both authors read and approved the final manuscript.

## Supplementary Material

Additional file 1 **Completeness of plant myosin sequences.** As indicator of the completeness of the assembled plant myosin sequences from whole genome projects the lengths of the myosin motor domains and the full-length proteins are listed. Based on the length difference to the supposed length of the full-length sequence, proteins are classified as “complete”, “partial” (up to 5% of the sequence missing) and “fragment” (more then 5% of the sequence missing). Only “complete” and “partial” sequences were used for phylogenetic tree calculations. Protein names consist of the species abbreviation, protein class and subtype designation as used throughout this analysis. Click here for file

Additional file 2 **Analysis of alternative splice variants.** The file contains an in-depth analysis of reported alternative splice variants of plant myosins. We could not find any support for alternative splice variants in the available cDNA/EST data and present evidence that the reported cases are rather examples of incompletely spliced transcripts. Click here for file

Additional file 3 **Phylogenetic trees of the plant myosins based on the motor domain and full-length sequences.** The grouping of plant myosin sequences into different subtypes is based on the phylogenetic trees calculated with RAxML (bootstrap values in percentages), MrBayes (posterior probabilities) and ClustalW (bootstrap values as absolute occurrences of branchings in 1,000 trees). Different subsets of the myosin sequences (the alignment is available in Additional file 8) were used to calculated the trees: the myosin head domains of representative sequences with less than 90% sequence identity (as obtained by CD-Hit), the myosin head domains of all complete myosin sequences included in this analysis, and the full-length sequences of representative sequences below an 90% identity threshold (CD-Hit). All resulting trees are included in this file. The protein abbreviation “Myo” was omitted in phylogenetic trees due to length limitations of sequence names in tree reconstruction software. Click here for file

Additional file 4 **Plant myosin classification.** Maximum-likelihood topology generated under the JTT + Γ model in RAxML showing branch lengths for the motor domains of 694 ingroup class VIII and XI myosins and five class V outgroup myosins. In this tree, plant myosin subtypes and major taxons are indicated by colour. The same phylogenetic tree is also included in Additional file 3. Click here for file

Additional file 5 **Gene structure conservation.** The file contains the independently created alignments of the gene structures of the class VIII and class XI myosins. The myosin gene structures are available from CyMoBase. The alignments were generated with GenePainter (http://www.motorprotein.de/genepainter.html, [72]). Click here for file

Additional file 6 **N-terminal SH3-like domain.** This figure shows the conservation of the N-terminal SH3-like domain. The alignment of all plant myosin N-terminal SH3-like domains is represented by a WebLogo and example sequences from *Arabidopsis thaliana* (*At*), *Homo sapiens* (*Hs*), *Drosophila melanogaster* (*Dm*) and *Caenorhabditis elegans* (*Ce*) are provided for orientation. Click here for file

Additional file 7 **Conserved motifs of class VIII myosins.** This figure shows the different conserved motifs of class VIII myosins. Each motif is represented by a WebLogo and example sequences from *Arabidopsis thaliana* (*At*), *Oryza sativa Indica group* (*Os_b*) and *Vitis vinifera* (*Vv*). Click here for file

Additional file 8 **Conserved motifs of class XI myosins.** The conserved C-terminal motifs of class XI myosins are shown as WebLogos together with examples of the *Arabidopsis thaliana* (*At*) myosins. The numbers given in front of each sequence indicate the amino acid positions in the respective sequences. Click here for file

Additional file 9 **Position of myosin genes on the chromosomes of *A. thaliana *and *O. sativa*.** The positions of the myosin genes on the genome are shown for all myosins encoded by the eudicot *Arabidopsis thaliana* and the monocot *Oryza sativa*. Click here for file

Additional file 10 **Myosin multiple sequence alignment.** Multiple sequence alignment of all plant myosins included in this analysis in FASTA format. Gaps at positions 372 and 1280 were included to indicate the myosin head domains used for most tree calculations. Click here for file

Additional file 11 **Phylogenetic trees.** Phylogeny of analysed plants based on myosin motor domain of all subtype 8A myosins. Trees were calculated with RAxML, MrBayes and ClustalW. RAxML and MrBayes provide support values as relative numbers, while ClustalW displayes absolute numbers (total 1,000 bootstraps). Click here for file

Additional file 12 **Detection of additionally proposed whole genome duplications based on phylogenetic analyses.** Maximum-likelihood topology generated under the JTT + Γ model in FastTree (1,000 replicates) showing branch lengths for the respective myosin motor domains. The topologies reveal evidence for further whole genome dupliations in *Nicotiana benthamiana* (*Nb*), *Manihot esculenta* (*Me*) and *Phoenix dactylifera* (*Phd*). **A) ***Solanum tuberosum* (*St*), *Solanum pimpinellifolium* (*Sop*) and *Lycopersicon esculentum* (*Le*). **B) ***Jatropha curcas* (*Jc*) and *Ricinus communis* (*Ric*). **C) ***Musa acuminata* (*Mua*) and *Oryza sativa (indica cultivar)* (*Os_b*). Click here for file

Additional file 13 **Species and genome assembly information.** This file contains the full taxonomy and the source of the genome assembly data for all species analysed. Genome analyses are referenced if these have already been published. All information is also available at CyMoBase (http://www.cymobase.org, [24]). Click here for file
