# Supplementary material for: Whole genome duplication events in plant evolution reconstructed and predicted using myosin motor proteins
Source: BMC Evol Biol. 2013 Sep 22;13:202. doi: 10.1186/1471-2148-13-202 (PMC3850447; doi:10.1186/1471-2148-13-202)
Supplement: Additional file 2 — Analysis of alternative splice variants. The file contains an in-depth analysis of reported alternative splice variants of plant myosins. We could not find any support for alternative splice variants in the available cDNA/EST data and present evidence that the reported cases are rather examples of incompletely spliced transcripts. [file 1471-2148-13-202-S2.pdf]

Alternatively spliced isoforms of plant myosins

Several alternative splice forms have been reported for *Oryza* and *Arabidopsis* myosins [1, 2]. Accordingly, the *Oryza* myosin-8A could be spliced into three isoforms, two derived from cDNA and one from predictions [1]. The predicted sequence lacked a 100% conserved short exon, XLLEK, which codes for the S7B strand and the following bulge [3] and is located directly next to switch-I [4]. This proposed isoform contained further sequence within the motor domain, which, in comparison with our sequence, has been mispredicted as exonic from intronic sequence. The cDNA sequences suggested two alternatively spliced forms for the C-terminal domain. In one of the cDNA clones (GenBankID AY374513), the last two exons of the *Os*Myo8A gene would be spliced by alternative 3' and 5' splice sites. However, such a sequence would require splicing of the intron with the junctions GA---AT, a combination, which does not exist in mammalian genomes according to an analysis of non-canonical splice sites [5, 6] and, although not listed in detail, is highly likely to not be present in *Arabidopsis* [7] (Figure 1). To our knowledge, non-canonical GA donor sites have been reported only rarely [8, 9] and only in combination with an AG acceptor site. In the other cDNA clone (GenBankID AY374514), a sequencing error led to a frame shift and accordingly a different C-terminal sequence, which has been misinterpreted as differently spliced isoform. Although millions of EST clones from plants are available we could not find any support for the suggested alternative splice variants of the class VIII myosins. Explicitly, we could not find any evidence for alternative splicing of the coding exons of any class VIII myosins.

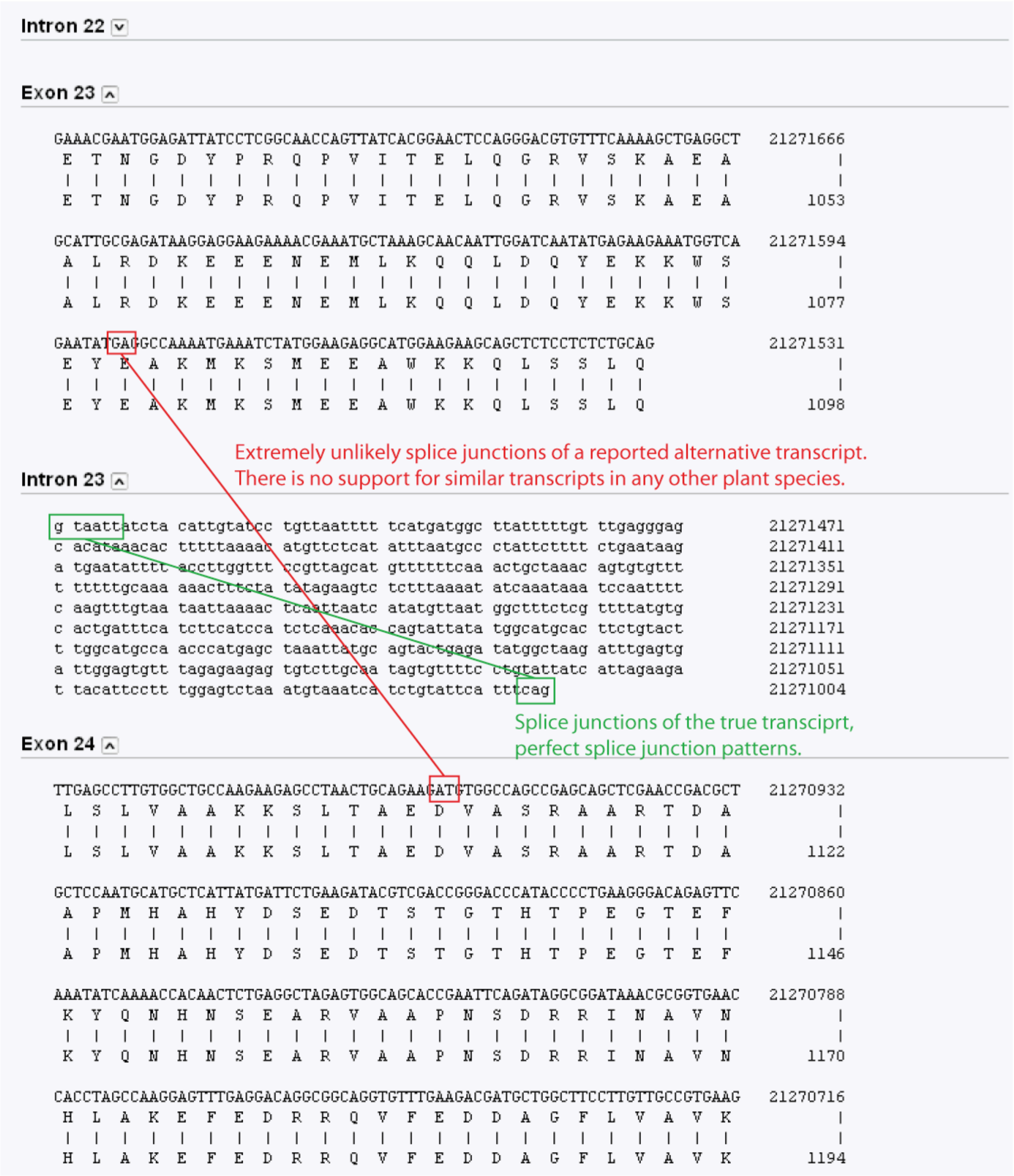

Figure 1: Section of the *Os*Myo8A gene, showing the region from exon 23 to exon 24.

Three alternative isoforms have been reported for the *Os*Myo11H gene [1], which are characterised by the differential inclusion of exon 27 and intron retention of the introns 36, 37 and 38. Also, intron retention of intron 31 has been reported (GenBankID AY374516), although this is most probably a sequencing error as the two bases of the 5’ splice site of intron 31 are missing in the sequence. In the analysis of the available EST data, exclusion of exon 27 (and homologous exons in other

species) has only been found twice, in AY374517 and one EST clone of *Carica papaya* (EX299313, *Cip*Myo11H), and only for Myo11H subtypes. In all other Myo11 subtypes the respective exon is included. Also, intron retention of any of the mentioned introns is not supported by EST data. While exon 27 encodes part of the unique region between the coiled-coil region and the DIL domain and its exclusion would probably not disturb the structure of the myosin tail, intron retention of any of the subsequent introns would result in an incomplete and probably unstable DIL domain. Thus we propose, that transcripts excluding exon 27 in *Os*Myo11H and respective exons in other Myo11 homologs are minor, less expressed isoforms (if existing at all), and that transcripts retaining introns between exons coding for the DIL domain are the result of splicing errors and will not be translated.

In *Arabidopsis*, three isoforms have been reported for the Myo11E gene. One of the alternative splice forms is built from an alternative acceptor site through that the start position for the second exon is changed leading to the exclusion of the N-terminal SH3-like domain and the first few residues of the motor domain. It has been shown for the class II myosin from *Dictyostelium discoideum* that removal of the N-terminal SH3-like domain led to complete loss of myosin-2 function in vivo [10]. We guess that this is also the reason for the marginal expression of this transcript in *Arabidopsis* [2] and that this isoform is non-functional. The other alternative isoform of *At*Myo11E is characterised by retention of intron 26 [2]. This isoform has been found in substantial quantities in RT-PCR analyses, but we did not find any further case in the available EST data. Instead, retention of the corresponding intron 27 is found in two EST clones of *Citrus limonia*. In both cases putative intron retention results in premature stop codons and thus class XI myosins without DIL domains. In contrast to the putative intron retention events in *Os*Myo11H, intron retention of

either intron 26 or intron 27 would not lead to structural disturbed proteins. However, the sequences within the retained introns up to the premature stop codons are not conserved between even closely related species and would lead to putative myosins with different C-termini and even potential myosins with DIL domains if the retained intron sequence does not encode a stop codon. Because of the lack of conservation, such splice variants are either specific to the *At*Myo11E gene or rarely expressed minor isoforms. Together, in contrast to the many alternative splice variants found for the class V myosins in vertebrates, which have an identical domain architecture [11], class VIII and class XI myosins in plants are not alternatively spliced or alternatively spliced isoforms represent negligible isoforms.

References

1. Jiang S, Ramachandran S: **Identification and molecular characterization of myosin gene family in Oryza sativa genome**. *Plant Cell Physiol.* 2004, **45**:590–599.

2. Peremyslov VV, Mockler TC, Filichkin SA, Fox SE, Jaiswal P, Makarova KS, Koonin EV, Dolja VV: **Expression, Splicing, and Evolution of the Myosin Gene Family in Plants**. *Plant Physiol.* 2011, **155**:1191–1204.

3. Cope MJ, Whisstock J, Rayment I, Kendrick-Jones J: **Conservation within the myosin motor domain: implications for structure and function**. *Structure* 1996, **4**:969–987.

4. Rayment I, Rypniewski WR, Schmidt-Bäse K, Smith R, Tomchick DR, Benning MM, Winkelmann DA, Wesenberg G, Holden HM: **Three-dimensional structure of myosin subfragment-1: a molecular motor**. *Science* 1993, **261**:50–58.

5. Burset M, Seledtsov IA, Solovyev VV: **Analysis of canonical and non-canonical splice sites in mammalian genomes**. *Nucleic Acids Res.* 2000, **28**:4364–4375.

6. Burset M, Seledtsov IA, Solovyev VV: **SpliceDB: database of canonical and non-canonical mammalian splice sites**. *Nucleic Acids Res.* 2001, **29**:255–259.

7. Alexandrov NN, Troukhan ME, Brover VV, Tatarinova T, Flavell RB, Feldmann KA: **Features of Arabidopsis genes and genome discovered using full-length cDNAs**. *Plant Mol. Biol.* 2006, **60**:69–85.

8. Twigg SR, Burns HD, Oldridge M, Heath JK, Wilkie AO: **Conserved use of a non-canonical 5' splice site (/GA) in alternative splicing by fibroblast growth factor receptors 1, 2 and 3**. *Hum. Mol. Genet.* 1998, **7**:685–691.

9. Bradley KJ, Cavaco BM, Bowl MR, Harding B, Young A, Thakker RV: **Utilisation of a cryptic non-canonical donor splice site of the gene encoding PARAFIBROMIN is associated with familial isolated primary hyperparathyroidism**. *J. Med. Genet.* 2005, **42**:e51.

10. Fujita-Becker S, Tsiavaliaris G, Ohkura R, Shimada T, Manstein DJ, Sutoh K: **Functional characterization of the N-terminal region of myosin-2**. *J. Biol. Chem.* 2006, **281**:36102–36109.

11. Lambert J, Naeyaert JM, Callens T, De Paepe A, Messiaen L: **Human Myosin V Gene Produces Different Transcripts in a Cell Type-Specific Manner**. *Biochemical and Biophysical Research Communications* 1998, **252**:329–333.
