# Supplementary figures and images for: Whole genome duplication events in plant evolution reconstructed and predicted using myosin motor proteins
Source: BMC Evol Biol. 2013 Sep 22;13:202. doi: 10.1186/1471-2148-13-202 (PMC3850447; doi:10.1186/1471-2148-13-202)

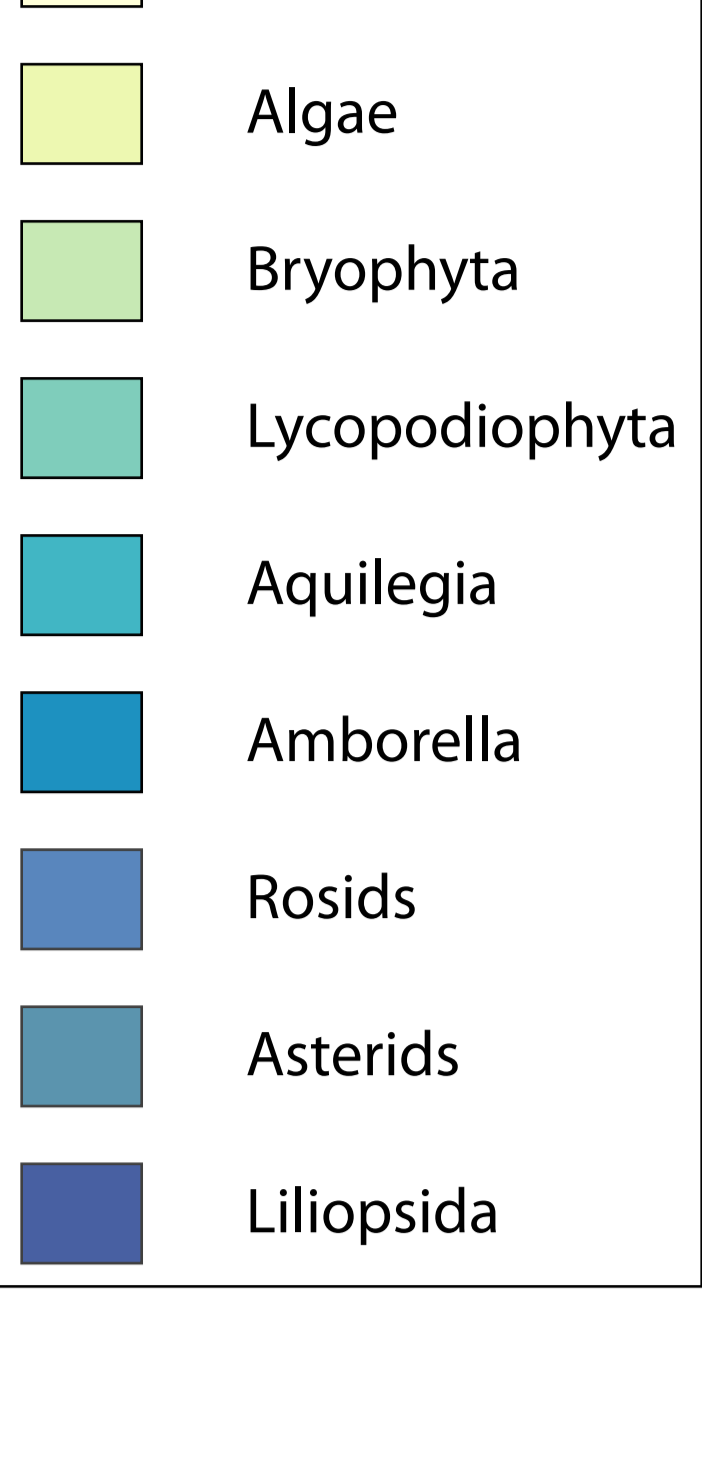

Class V myosin (outgroup)

8B

8A

11G

11H

11A

11B

11E

11F

11E

11C

11D

11C

Supplement: Additional file 4 — Plant myosin classification. Maximum-likelihood topology generated under the JTT + Γ model in RAxML showing branch lengths for the motor domains of 694 ingroup class VIII and XI myosins and five class V outgroup myosins. In this tree, plant myosin subtypes and major taxons are indicated by colour. The same phylogenetic tree is also included in Additional file 3. [file 1471-2148-13-202-S4.pdf]

**A** Arabidopsis thaliana

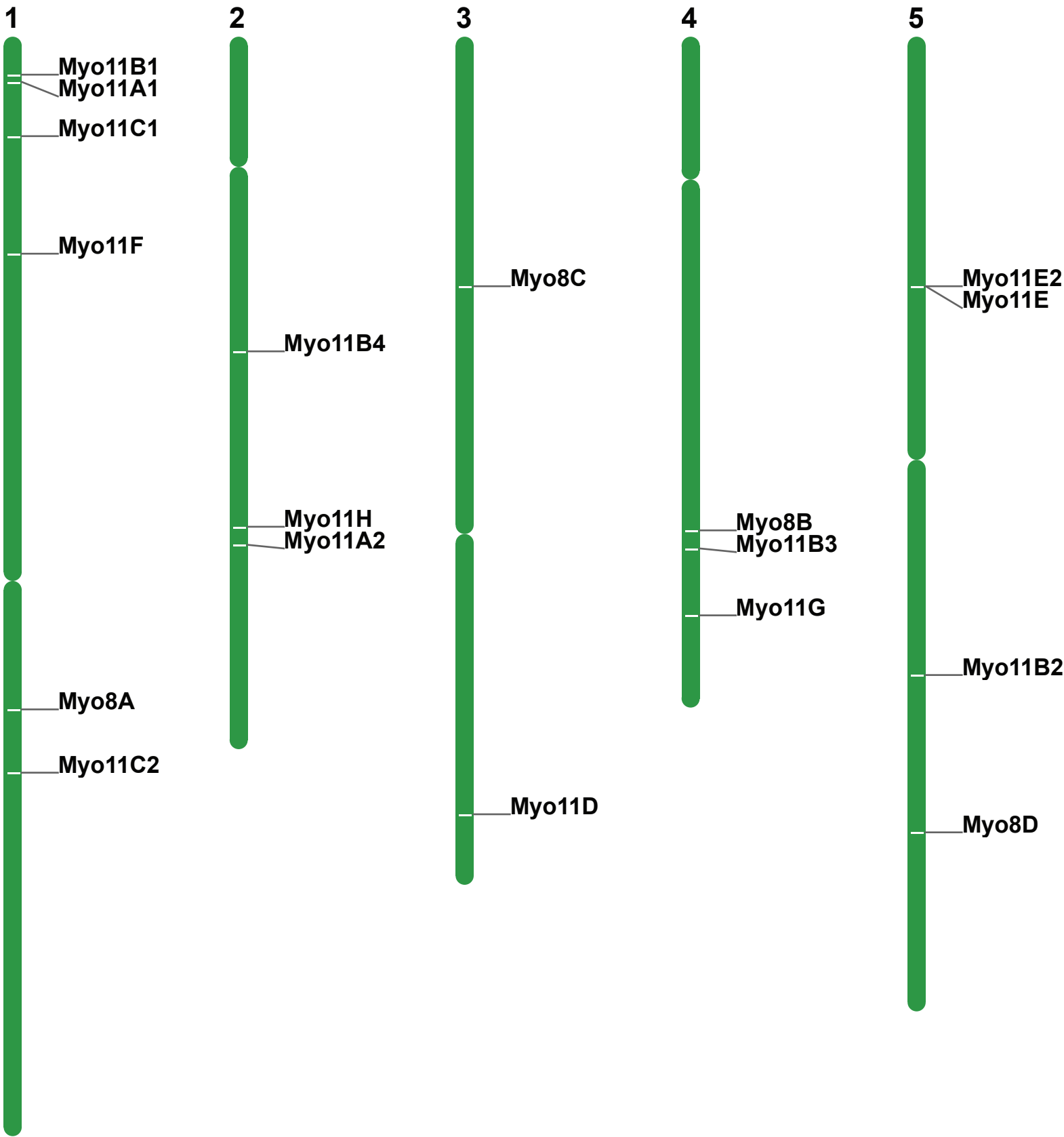

**B** Orzya sativa Japonica group

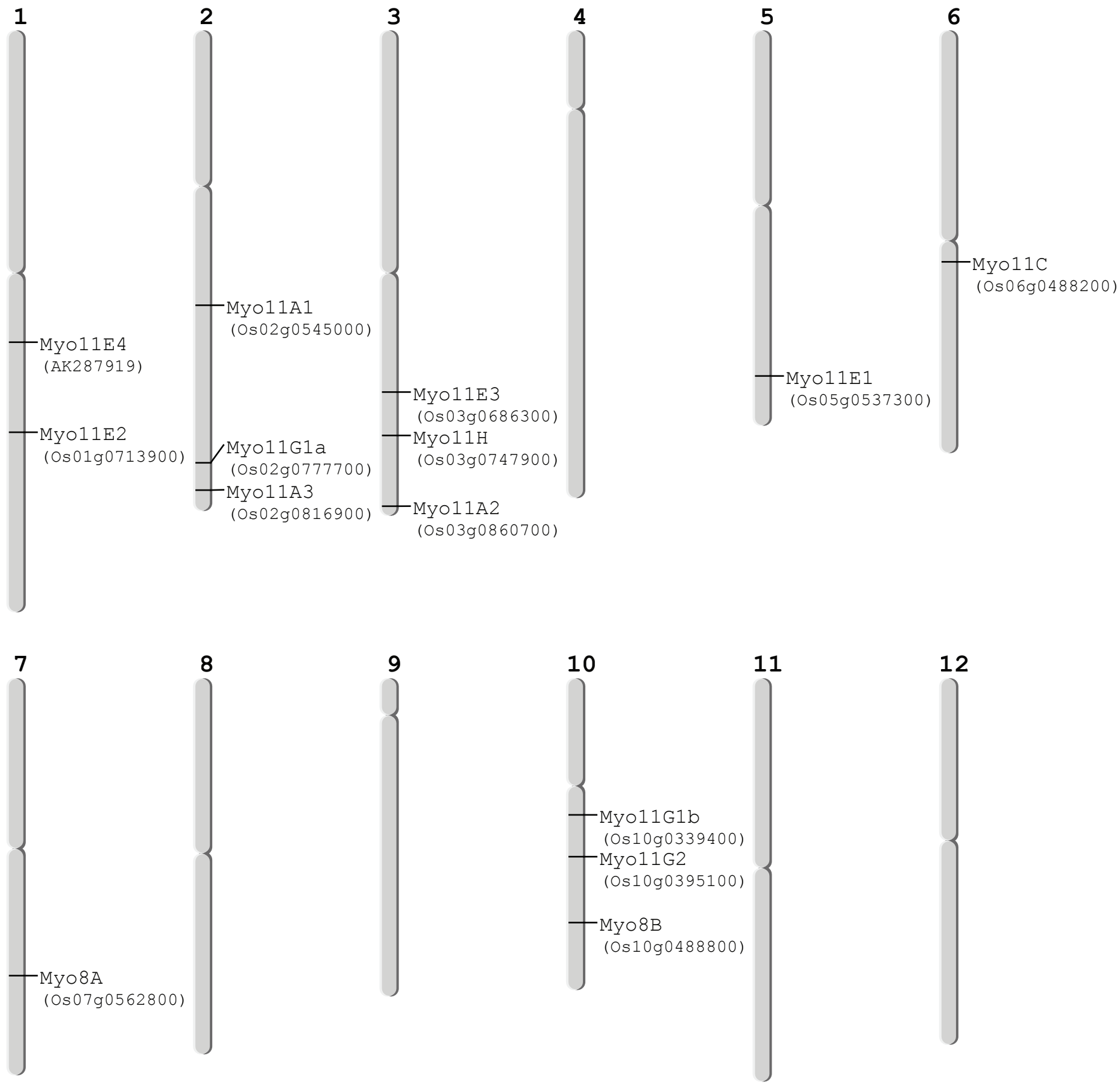

Supplement: Additional file 9 — Position of myosin genes on the chromosomes of A. thaliana and O. sativa. The positions of the myosin genes on the genome are shown for all myosins encoded by the eudicot Arabidopsis thaliana and the monocot Oryza sativa. [file 1471-2148-13-202-S9.pdf]

# MrBayes

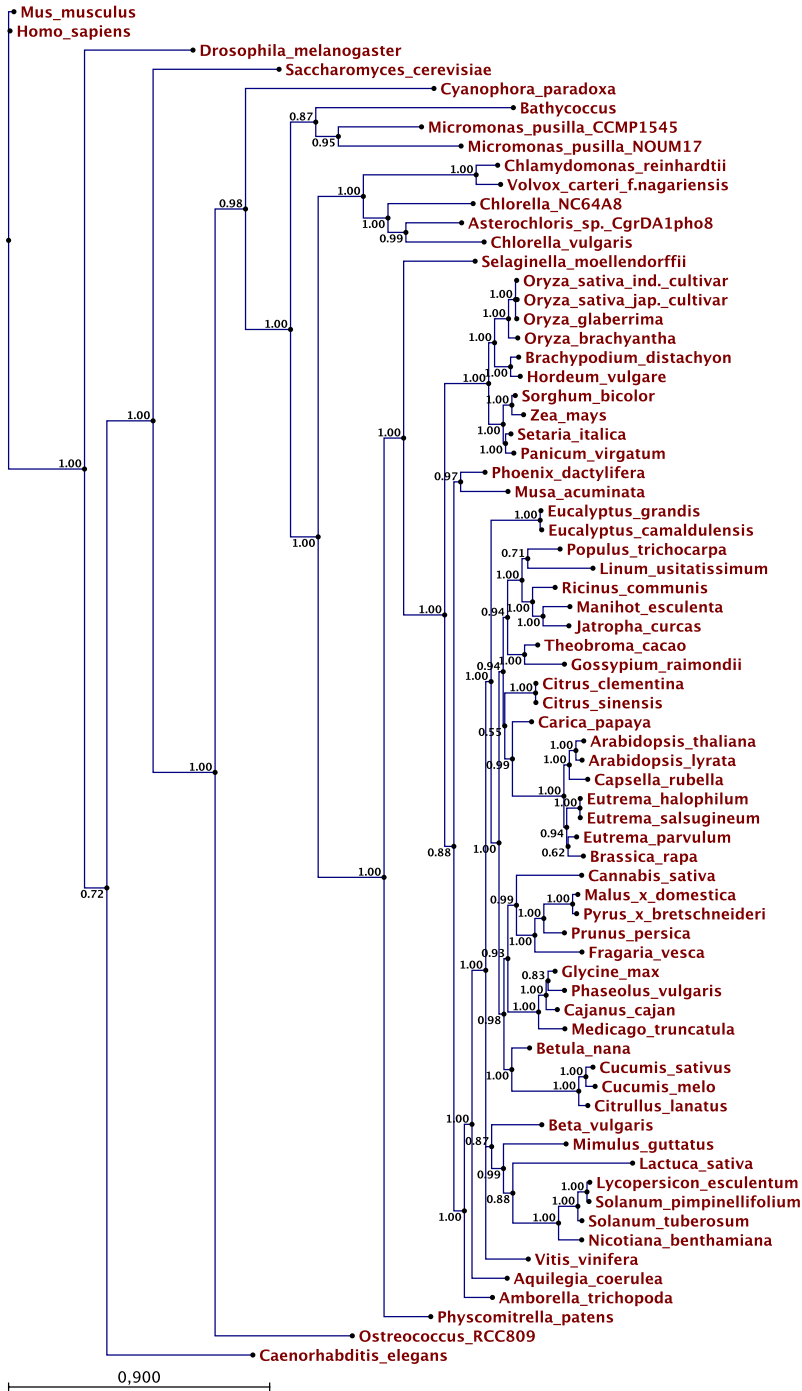

# ClustalW

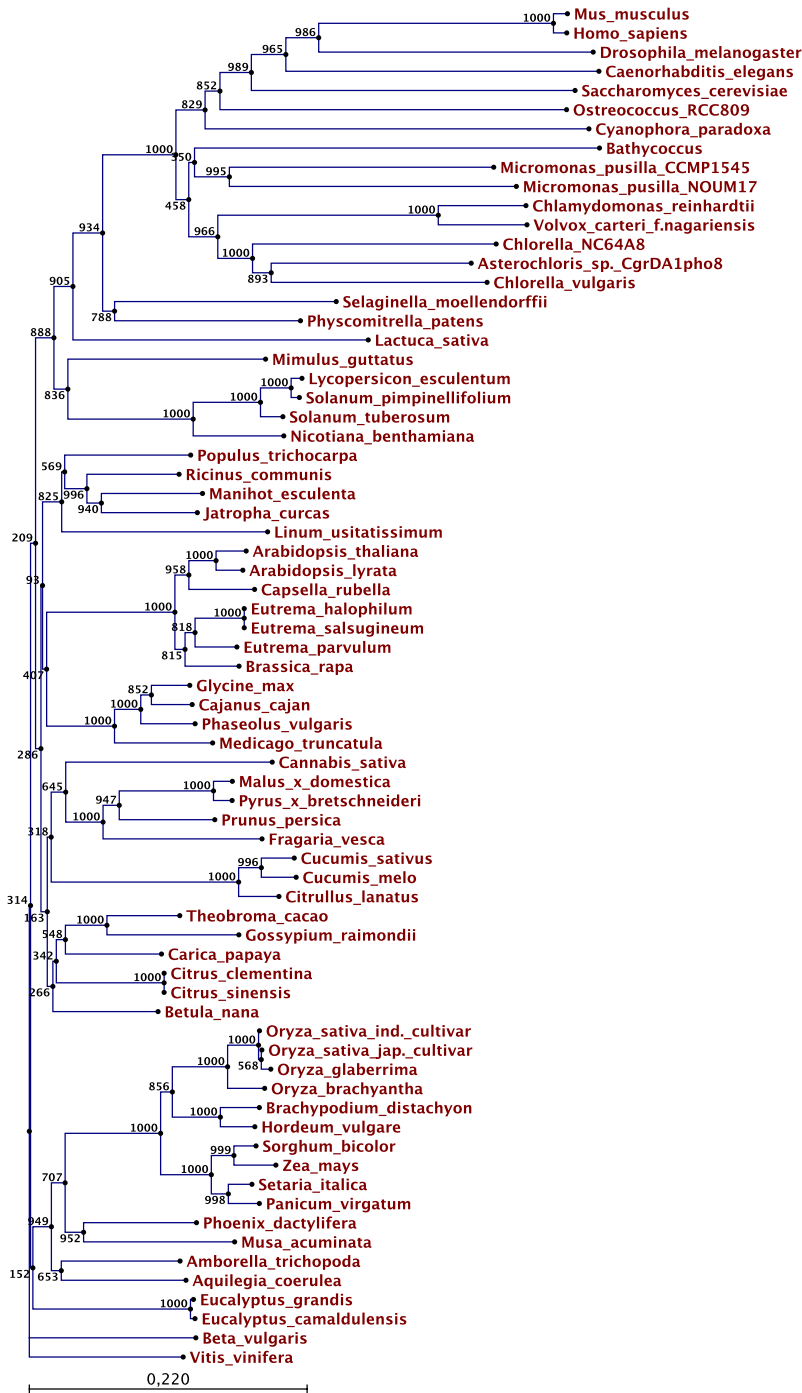

# RxML

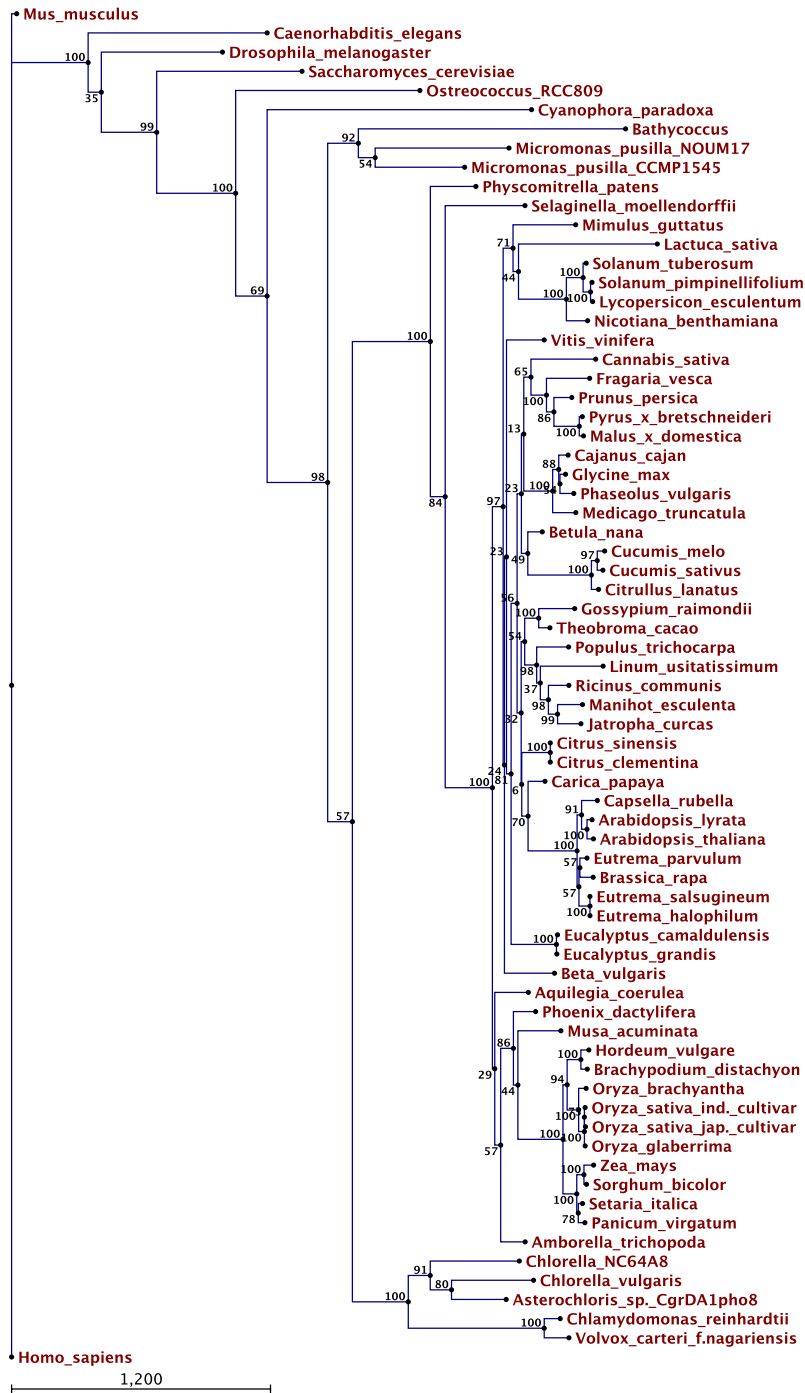

Supplement: Additional file 11 — Phylogenetic trees. Phylogeny of analysed plants based on myosin motor domain of all subtype 8A myosins. Trees were calculated with RAxML, MrBayes and ClustalW. RAxML and MrBayes provide support values as relative numbers, while ClustalW displayes absolute numbers (total 1,000 bootstraps). [file 1471-2148-13-202-S11.pdf]

A

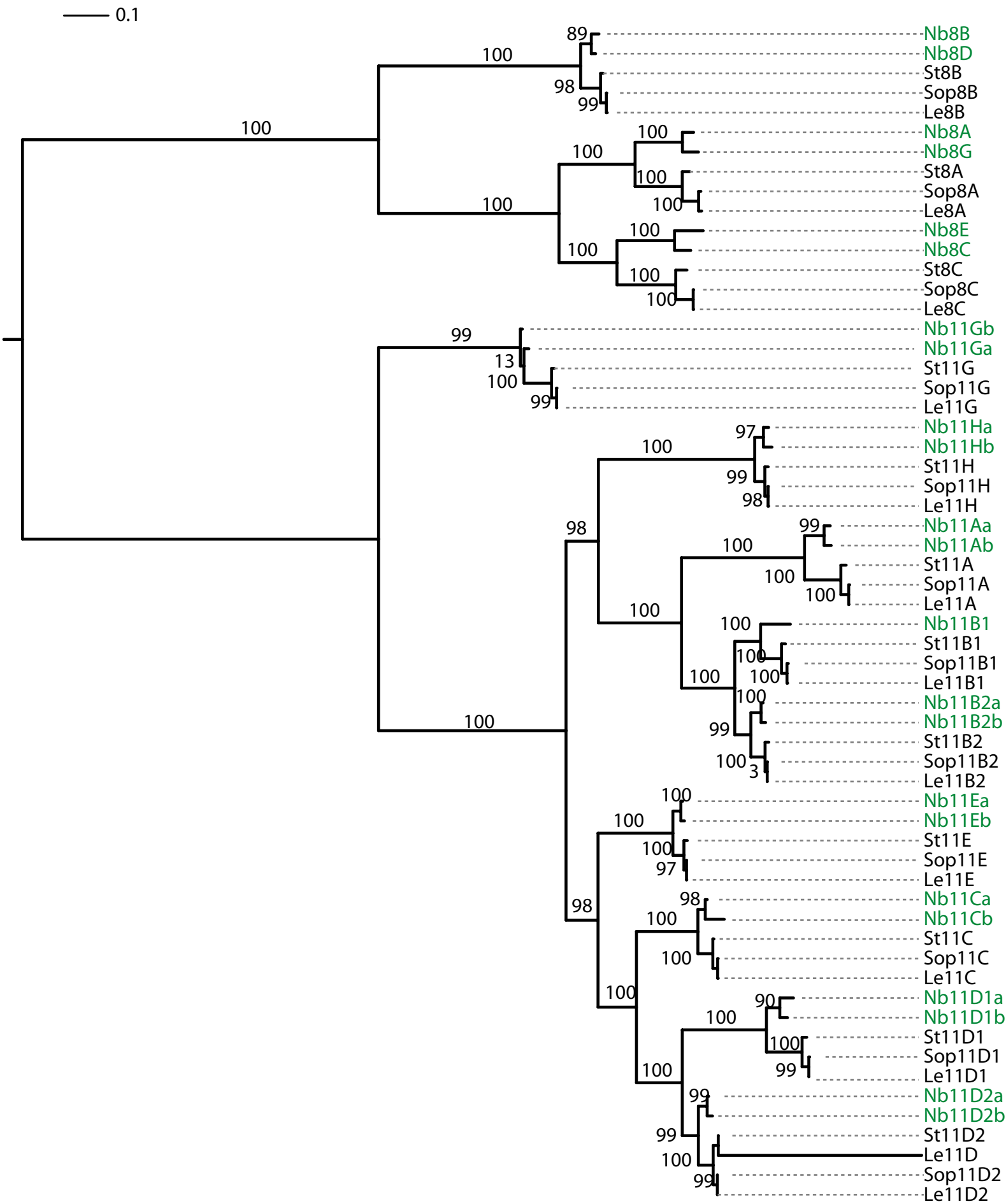

B

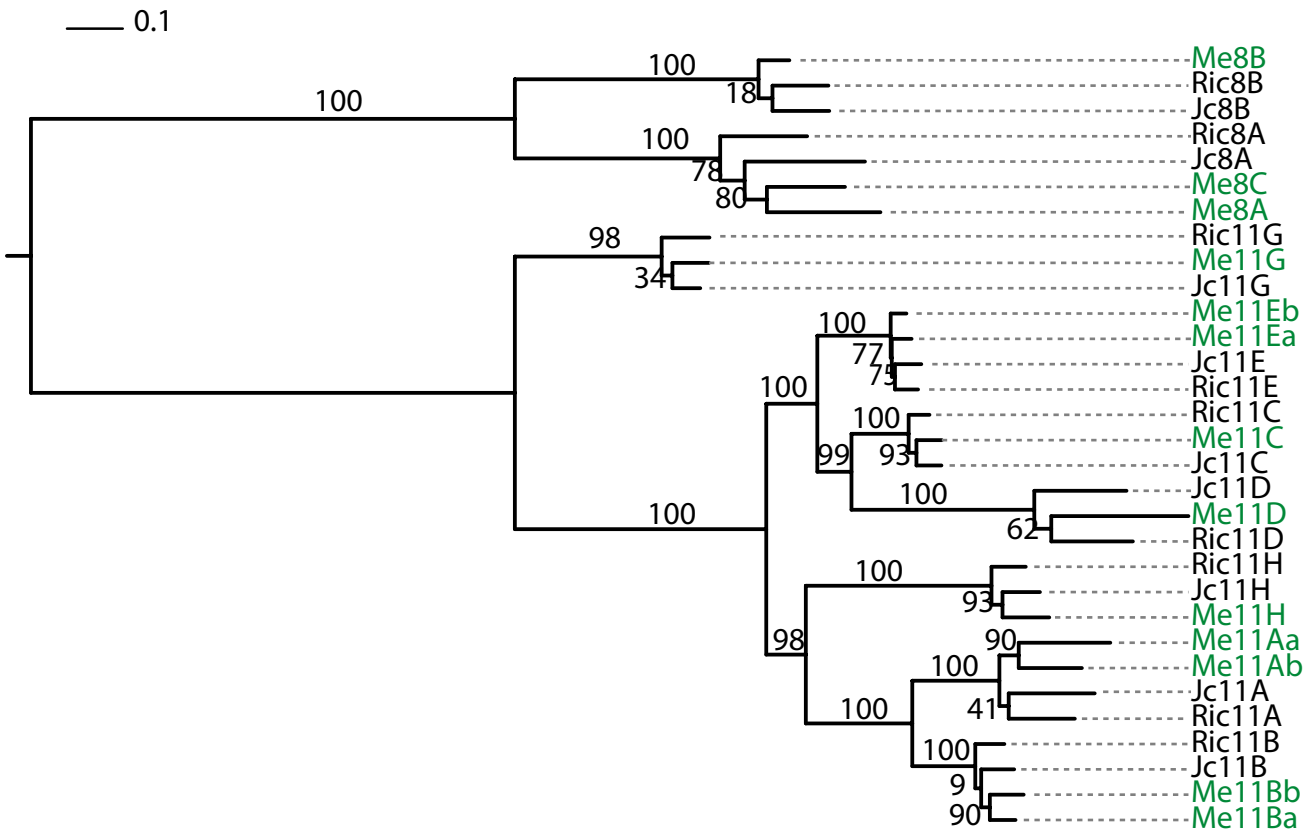

C

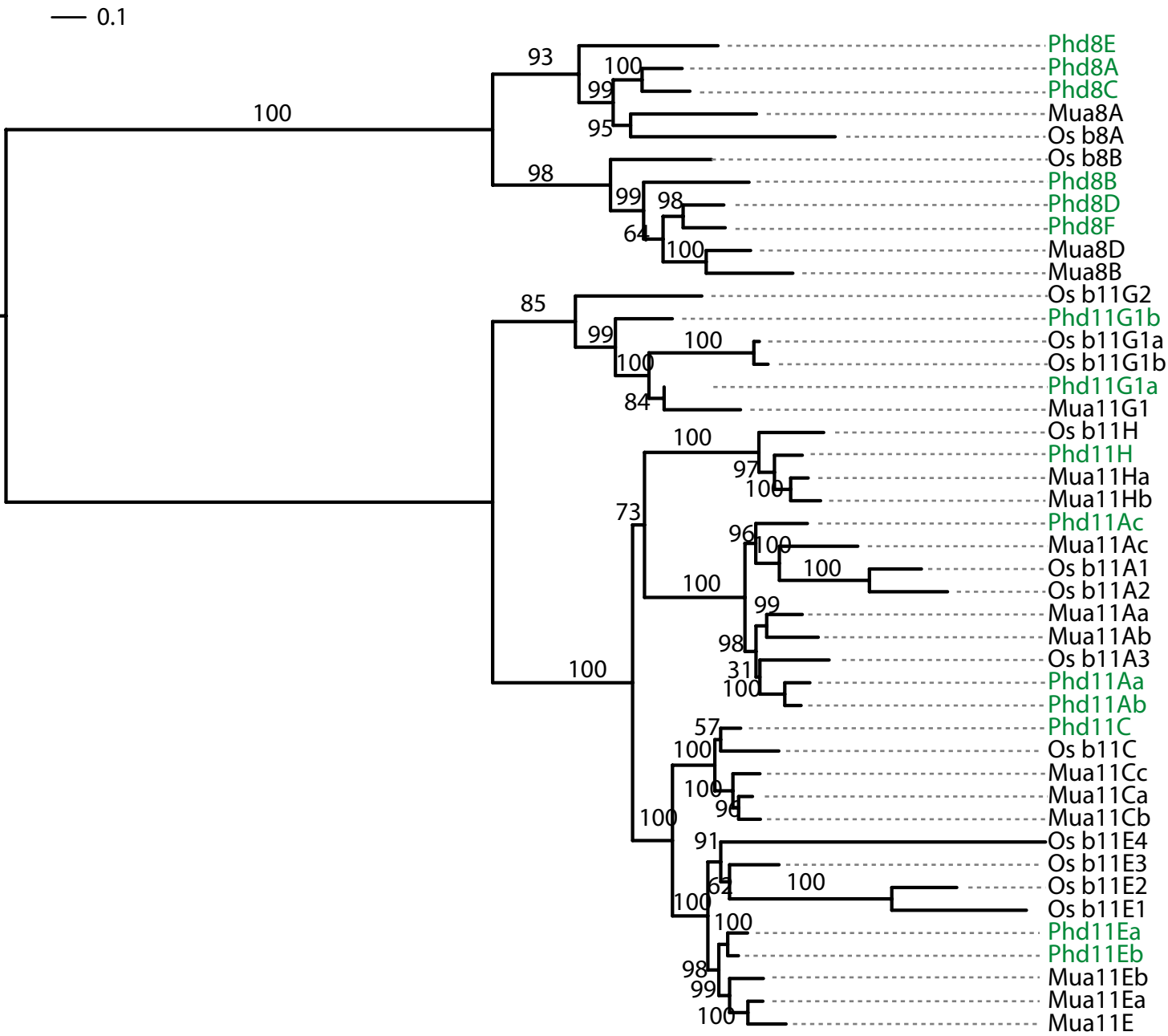

Supplement: Additional file 12 — Detection of additionally proposed whole genome duplications based on phylogenetic analyses. Maximum-likelihood topology generated under the JTT + Γ model in FastTree (1,000 replicates) showing branch lengths for the respective myosin motor domains. The topologies reveal evidence for further whole genome dupliations in Nicotiana benthamiana (Nb), Manihot esculenta (Me) and Phoenix dactylifera (Phd). A) Solanum tuberosum (St), Solanum pimpinellifolium (Sop) and Lycopersicon esculentum (Le). B) Jatropha curcas (Jc) and Ricinus communis (Ric). C) Musa acuminata (Mua) and Oryza sativa (indica cultivar) (Os_b). [file 1471-2148-13-202-S12.pdf]
