# Supplementary material for: Whole genome duplication events in plant evolution reconstructed and predicted using myosin motor proteins
Source: BMC Evol Biol. 2013 Sep 22;13:202. doi: 10.1186/1471-2148-13-202 (PMC3850447; doi:10.1186/1471-2148-13-202)
Supplement: Additional file 6 — N-terminal SH3-like domain. This figure shows the conservation of the N-terminal SH3-like domain. The alignment of all plant myosin N-terminal SH3-like domains is represented by a WebLogo and example sequences from Arabidopsis thaliana (At), Homo sapiens (Hs), Drosophila melanogaster (Dm) and Caenorhabditis elegans (Ce) are provided for orientation. [file 1471-2148-13-202-S6.pdf]

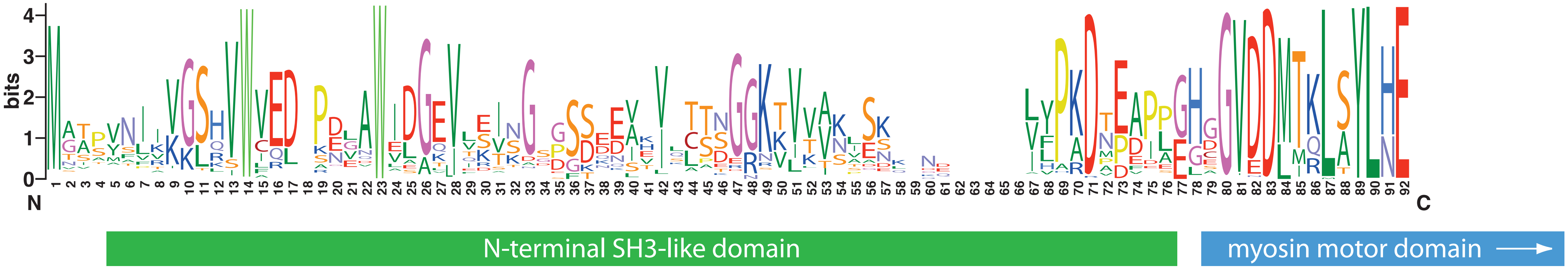


```
HsMyo5A     1  MAASELYTKFARVWIPD-PEEVWKSAELLKDYKPG-DKVLLLHLEEG-KDLEYHLDPKTKE-----LPHLRNPDIL--VGENDLTALSYLHE
CeMyo5     12  LFPLQNFKKGVRIWHRH-PTLVWIGATLEEDITFQ-TRNVRIRLEDD-TEVEYAIKSLDQ------LPFLRNPAFL--VGKDDLTLLSYLHE
DmMyo5      2  SSEEMLYAQGAKIWVPH-ADLVWESATLEESYR---KGAGFLKICTD-SGKLKEVKLKADGSD---LPPLRNPAIL--VGQNDLTTLSYLHE
AtMyo8A   104  -SAEYFVREKLCVWCRVAANGQWHLGKIHSTSSSD--DVCVMLSAND-DVVKVAMEE---------IFPA-NPEIL--EGVEDLTQLSYLNE
AtMyo8C   154  -NVEYFIKKKLRVWCRV-SNGQWQLGKIQSTSA----DTSLVMLSTA-NVVKVSTEE---------LFPA-NPDIL--EGVEDLIQLSYLNE
AtMyo8B    98  -TNVYARKKVLQFWVQL-PNGNWELGKIMSTSG----EESVIVVTEG-KVLKVKSET---------LVPA-NPDIL--DGVDDLMQLSYLNE
AtMyo8D   106  -TSAYARKKILQSWIQL-PNGNWELGKILSTSG----EESVISLPEG-KVIKVISET---------LVPA-NPDIL--DGVDDLMQLSYLNE
AtMyo11C1   1  MGTPVNIIVGSHVWFED-PEVAWIDGEVEKING----QEVVIQATTG-KKVTAKLSK---------IYPKDVEAPA--GGVDDMTKLSYLHE
AtMyo11C2   1  MGTPVNIIVGSHVWIED-SDVAWIDGLVEKING----QDVEVQATNG-KKITAKLSK---------IYPKDMEAPA--GGVDDMTKLSYLHE
AtMyo11D    1  --MAENIMVDSHVWVED-PERAWIDGVVLNIKG----EEAEIKTNDG-RDVIANLSR---------LYPKDTEAPS--EGVEDMTRLSYLHE
AtMyo11B1   1  MVATFNPAVGSHVWVED-PDEAWLDGEVVEING----DQIKVLCASG-KQVVVKDSN---------IYPKDVEAPA--SGVEDMTRLAYLHE
AtMyo11B2   1  MVANFNPSVGSFVWVED-PDEAWIDGEVVQVNG----DEIKVLCTSG-KHVVTKISN---------AYPKDVEAPA--SGVDDMTRLAYLHE
AtMyo11A1   1  MAASAKVTVGSHVWVED-PDDAWIDGEVEEVNS----EEITVNC-SG-KTVVAKLNN---------VYPKDPEFPE--LGVDDMTKLAYLHE
AtMyo11A2   1  -MASVKVTVGSQVWVED-PDEAWLDGEVVEANG----QEIKVNCQ-T-KTVVAKVNA---------VHPKDPEFPE--LGVDDMTKLAYLHE
AtMyo11B3   1  -MACTTVNVGSCVWVED-PEVAWIDGEVIEVKG----SDIKVKCTSG-KTVAIKVSS---------AYPKDVEAPA--SGVDDMTRLAYLHE
AtMyo11B4   1  -MACSTVKVGSIVWVQD-PEEAWIDGEVVEVNG----EDIKVQCTSG-KTVVAKGSN---------TYPKDMEVPP--SGVDDMTTLAYLHE
AtMyo11H    1  MGTPVNITLGSHVWVED-PELAWISGEVTEIKG----TNAKIVTANG-KTVVASISS---------IYPKDTEAPP--AGVDDMTKLAYLHE
AtMyo11G    5  LPMELNLRKGDKVWVED-KDLAWIAADVLDSFD----NKLHVETSTG-KKVFVSPEK---------LFRRDPDDEE-HNGVDDMTKLTYLHE
AtMyo11E    1  MVGPVNIIVGSHVWIED-PGAAWIDGEVVKING----EEVHAHTTNG-KTVVANIAN---------VFPKDTEAPP--GGVDDMTKLSYLHE
AtMyo11F    1  MAAPV-IIVGSHVWVED-PHLAWIDGEVTRIDG----INVHVKTKKG-KTVVTNV-----------YFPKDTEAPS--GGVDDMTKLSYLHE
```
