# Supplementary material for: Whole genome duplication events in plant evolution reconstructed and predicted using myosin motor proteins
Source: BMC Evol Biol. 2013 Sep 22;13:202. doi: 10.1186/1471-2148-13-202 (PMC3850447; doi:10.1186/1471-2148-13-202)
Supplement: Additional file 7 — Conserved motifs of class VIII myosins. This figure shows the different conserved motifs of class VIII myosins. Each motif is represented by a WebLogo and example sequences from Arabidopsis thaliana (At), Oryza sativa Indica group (Os_b) and Vitis vinifera (Vv). [file 1471-2148-13-202-S7.pdf]

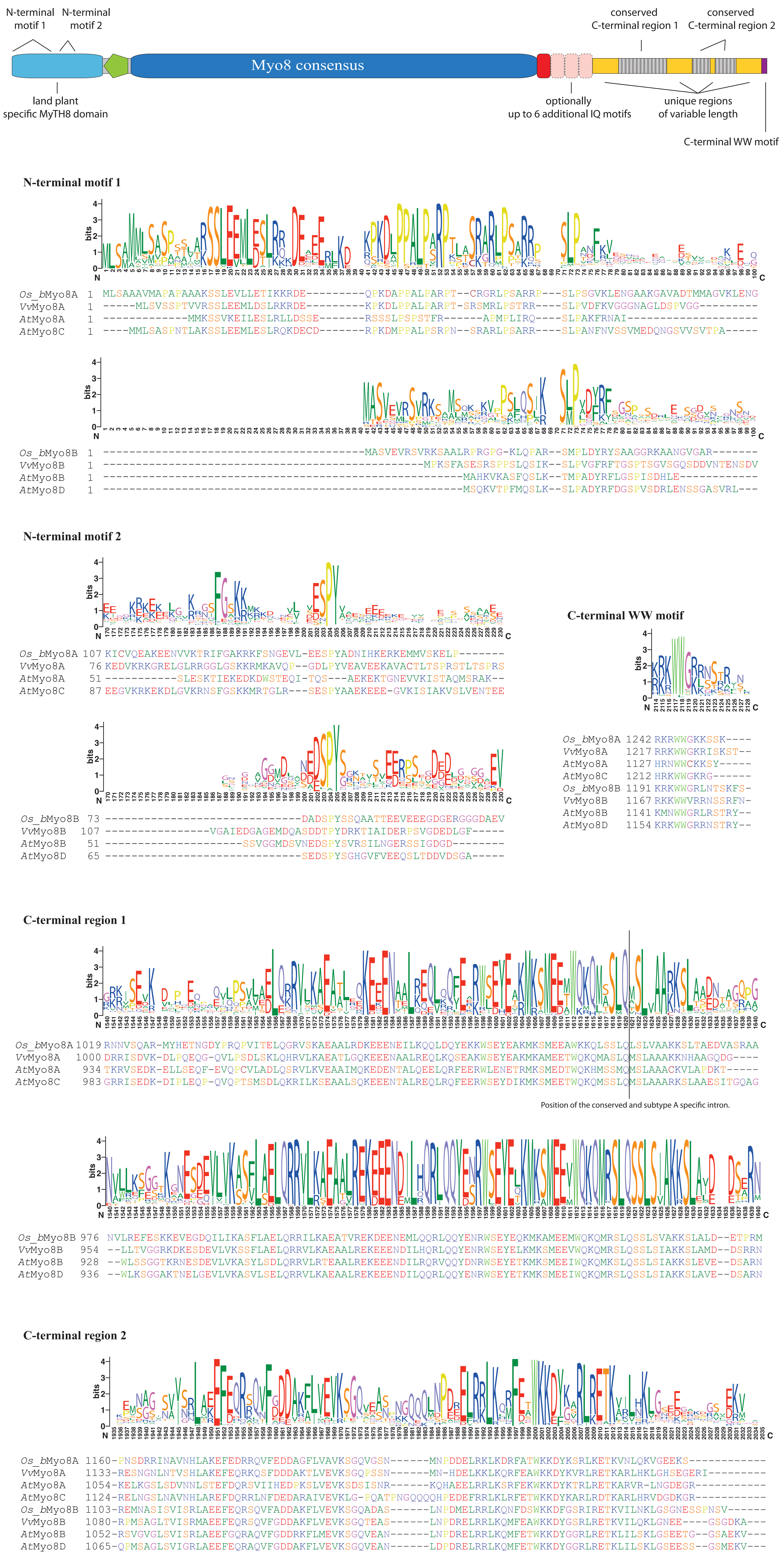

N-terminal motif 1
N-terminal motif 2
conserved C-terminal region 1
conserved C-terminal region 2
Myo8 consensus
land plant specific MyTH8 domain
optionally up to 6 additional IQ motifs
unique regions of variable length
C-terminal WW motif
N-terminal motif 1
Os_bMyo8A 1 MLSAAAVMAPAPAAAKSSLEVLLETIKKRDE---------QPKDAPPALPARPT--CRGRLPSARRP---SLPSGVKLENGAAKGAVADTMMAGVKLENG
VvMyo8A 1 -----MLSVSSPTVVRSSLEEMLDSLRKRDE---------KPKDLPPALPARPT-SRSMRLPSTRR----SLPVDFKVGGGNAGLDSPVGG---------
AtMyo8A 1 -------------MMKSSVKEILESLRLLDSSE-------RSSSLPSPSTFR------APMPLIRQ----SLPAKFRNAI--------------------
AtMyo8C 1 ----MMLSASPNTLAKSSLEEMLESLRQKDECD-------RPKDMPPALPSRPN--SRARLPSARR----SLPANFNVSSVMEDQNGSVVSVTPA-----
Os_bMyo8B 1 ---------------------------------------MASVEVRSVRKSAALRPRGPG-KLQPAR--SMPLDYRYSAAGGRKAANGVGAR-------
VvMyo8B 1 --MPKSFASESRSPPSLQSIK--SLPVGFRFTGSPTSGVSGQSDDVNTENSDV
AtMyo8B 1 --MAHKVKASFQSLK--TMPADYRFLGSPISDHLE-----------
AtMyo8D 1 --MSQKVTPFMQSLK--SLPADYRFDGSPVSDRLENSSGASVRL---
N-terminal motif 2
Os_bMyo8A 107 KICVQEAKEENVVKTRIFGAKRKFSNGEVL-EESPYADNIHKERKEMMVSKELP-------
VvMyo8A 76 KEDVKRKGRELGLRRGGLGSKKRMKAVQP--GDLPYVEAVEEKAVACTLTSPRSTLTSPRS
AtMyo8A 51 -----------SLESKTIEKEDKDWSTEQI-TQS---AEKEKTGNEVVKISTAQMSRAK--
AtMyo8C 87 EEGVKRKEKDLGVKRNSFGSKKMRTGLR---SESPYAAEKEEE-GVKISIAKVSLVENTEE
Os_bMyo8B 73 ------------------------------DADSPYSSQAATTEEVEEEGDGERGGGDAEV
VvMyo8B 107 ----------------VGAIEDGAGEMDQASDDTPYDRKTIAIDERPSVGDEDLGF-----
AtMyo8B 51 ---------------------SSVGGMDSVNEDSPYSVRSILNGERSSIGDGD--------
AtMyo8D 65 ------------------------------SEDSPYSGHGVFVEEQSLTDDVDSGA-----
C-terminal WW motif
Os_bMyo8A 1242 RKRWWGKKSSK----
VvMyo8A 1217 RRKWWGKRISKST--
AtMyo8A 1127 HRNWWCKKSY-----
AtMyo8C 1212 HRKWWGKRG------
Os_bMyo8B 1191 KRKWWGRLNTSKFS-
VvMyo8B 1167 RKKWWVRRNSSRFN-
AtMyo8B 1141 KMNWWGRLRSTRY--
AtMyo8D 1154 KRKWWGRRNSTRY--
C-terminal region 1
Os_bMyo8A 1019 RNNVSQAR-MYHETNGDYPRQPVITELQGRVSKAEAALRDKEEENEILKQQLDQYEKKWSEYEAKMKSMEEAWKKQLSSLQLSLVAAKKSLTAEDVASRAA
VvMyo8A 1000 DRRISDVK-DLPQEQG-QVLPSDLSKLQHRVLKAEATLGQKEEENAALREQLKQSEAKWSEYEAKMKAMEETWQKQMASLQMSLAAAKKNHAAGQDG----
AtMyo8A 934 TKRVSEDK-ELLSEQF-EVQPCVLADLQSRVLKVEAAIMQKEDENTALQEELQRFEERWLENETRMKSMEDTWQKHMSSMQMSLAAACKVLAPDKT-----
AtMyo8C 983 GRRISEDK-DIPLEQP-QVQPTSMSDLQKRILKSEAALSQKEEENTALREQLRQFEERWSEYDIKMKSMEETWQKQMSSLQMSLAAARKSLAAESITGQAG
Position of the conserved and subtype A specific intron.
Os_bMyo8B 976 NVLREFESKKEVEGDQILIKASFLAELQRRILKAEATVREKDEENEMLQQRLQQYENRWSEYEQKMKAMEEMWQKQMRSLQSSLSVAKKSLALD--ETPRM
VvMyo8B 954 --LLTVGGRKDKESDEVLVKSSFLAELQRRVLKAEAALREKEEENDILHQRLQQYENRWSEYELKMKSMEEVWQKQMRSLQSSLSIAKKSLAMD--DSRRN
AtMyo8B 928 --WLSSGGTKRNESDEVLVKASYLSDLQRRVLRTEAALREKEEENDILRQRVQQYDNRWSEYETKMKSMEEIWQKQMKSLQSSLSIAKKSLEVE--DSARN
AtMyo8D 936 --WLKSGGAKTNELGEVLVKASVLSELQRRVLKAEAALREKEEENDILQQRLQQYENRWSEYETKMKSMEEIWQKQMRSLQSSLSIAKKSLAVE--DSARN
C-terminal region 2
Os_bMyo8A 1160-PNSDRRINAVNHLAKEFEDRRQVFEDDAGFLVAVKSGQVGSN------MNPDDELRKLKDRFATWKKDYKSRLKETKVNLQKVGEEKS-----------
VvMyo8A 1133-RESNGNLNTVSHLAKEFEQRKQSFDDDAKTLVEVKSGQPSSN------MN-HDELKKLKQRFEAWKKDYKVRLRETKARLHKLGHSEGERI---------
AtMyo8A 1054-KELKGSLSDVNNLSTEFDQRSVIIHEDPKSLVEVKSDSISNR------KQHAEELRRLKSRFEKWKKDYKTRLRETKARVR-LNGDEGR-----------
AtMyo8C 1124-RELNGSLNAVNHLAREFDQRRLNFDEDARAIVEVKLG-PQATPNGQQQQHPEDEFRRLKLRFETWKKDYKARLRDTKARLHRVDGDKGR-----------
Os_bMyo8B 1103-REMNASISVISRLAEEFEQRSQVFADDAKFLVEVKSGQADAS------LNPDMELRRLKQNFDSWKKDFGSRIRETKVILNKLGSGNESSPNSV------
VvMyo8B 1080-RPMSAGLTVISRMAEEFEQRSQVFGDDAKFLVEVKSGQTEAS------LNPDRELRRLKQMFEAWKKDYGSRLRETKVILQKLGNEE----GSGDKA---
AtMyo8B 1052-RSVGVGLSVISRLAEEFGQRAQVFGDDRKFLMEVKSGQVEAN------LNPDRELRRLKQMFETWKKDYGGRLRETKLILSKLGSEETG--GSAEKV---
AtMyo8D 1065-QPMSAGLSVIGRLAEEFEQRAQVFGDDAKFLVEVKSGQVEAN------LDPDRELRRLKQMFETWKKDYGGRLRETKLILSKLGSEESS--GSMEKV---
