# Supplementary material for: Whole genome duplication events in plant evolution reconstructed and predicted using myosin motor proteins
Source: BMC Evol Biol. 2013 Sep 22;13:202. doi: 10.1186/1471-2148-13-202 (PMC3850447; doi:10.1186/1471-2148-13-202)
Supplement: Additional file 8 — Conserved motifs of class XI myosins. The conserved C-terminal motifs of class XI myosins are shown as WebLogos together with examples of the Arabidopsis thaliana (At) myosins. The numbers given in front of each sequence indicate the amino acid positions in the respective sequences. [file 1471-2148-13-202-S8.pdf]

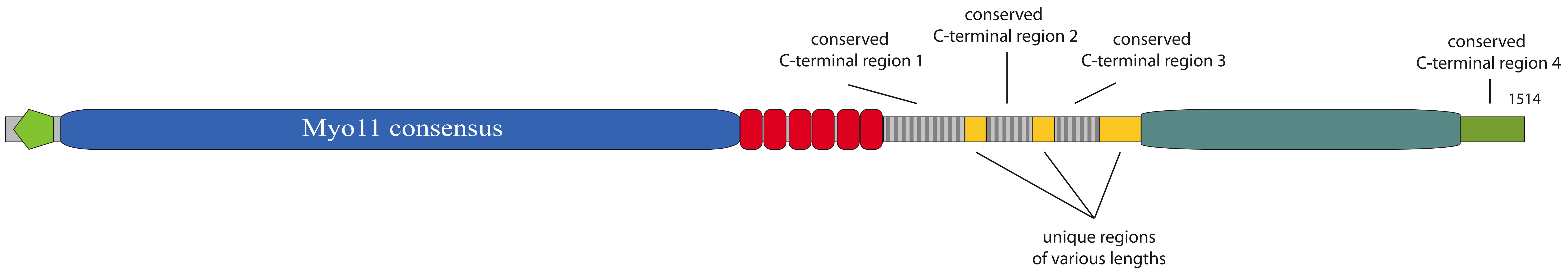

C-terminal region 1

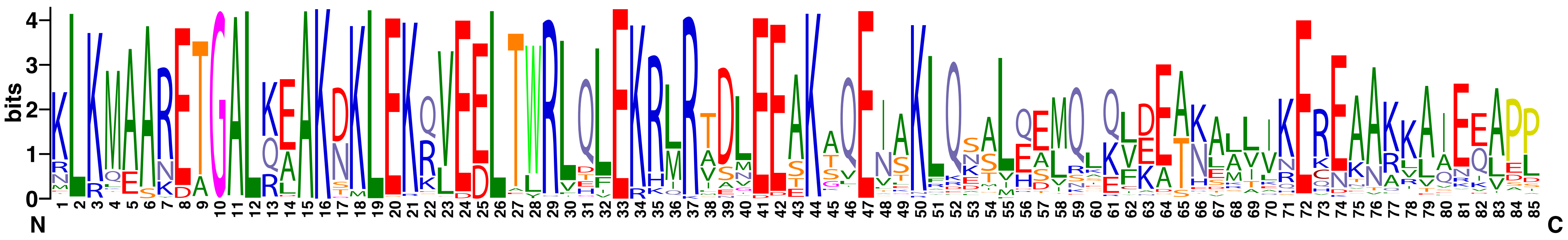

```
AtMyo11C1   876  KLKMAARETGALKEAKDMLEKKVEELTYRVQLEKRSRGDLEEAKTQEILKLKSSFEEMRKKVDETNALLLKEREAAKKAAEEAPP
AtMyo11C2   876  QLKMASRETGALKEAKDMLEKKVEELTYRAQLEKRSRVDLEEEKNQEIKKLQSSLEEMRKKVDETNGLLVKEREAAKKAIEEAPP
AtMyo11B1   876  NLKMAARETGALQEAKNKLEKQVEELTWRLQLEKRMRTDLEEAKKQENAKYESSLEEIQNKFKETEALLIKEREAAKTVSEVL--
AtMyo11B2   875  KLKMAARETGALQAAKNKLEKQVEELTWRLQLEKRIRTDLEEAKKQESAKAQSSLEELQLKCKETEALLIKEREAAKKIAETA--
AtMyo11B3   874  KLKMAAKETGVLEAAKSKLEKQVEELTWKLQLEKRMRTDMEESKTQENAKLRSALEEMQLQFKETKALHLQEVEAAKKMAETV--
AtMyo11B4   870  KLKTDARDTVVLQAAKSMLAEKVEELTWRLDLEKRMRVDMEVSKAQENAKLQLALEEIQLQFEETKVSLLKEVEAAKKTAAIV--
AtMyo11H    875  NLRMAARETGALKEAKDKLEKRVEELTWRLELEKNQKADLEDAKAQEIAKLQNNLTELQEKLDEAYAAIIRDKEAAKLAIEQAP-
AtMyo11G    881  KLKQVANEAGALRLAKTKLEKRLEDLEWRLQLEKRLRTSGEEAKSSEISKLQKTLESFSLKLDAARLATINECNKNAVLEKQLDI
AtMyo11E    876  TLKMAARDTGALREAKDKLEKRVEELTWRLQLEKRQRTELEEAKTQEYAKQQEALETMRLQVEEANAAVIREREAARKAIEEAP-
AtMyo11F    873  MLKMAARDTGALKDAKNKLEQRVEELSLRLHLEKRLRTDLEEAKVQEVAKLQEALHTMRLQLKETTAMVVKEQEAARVAIEEAS-
```

C-terminal region 2

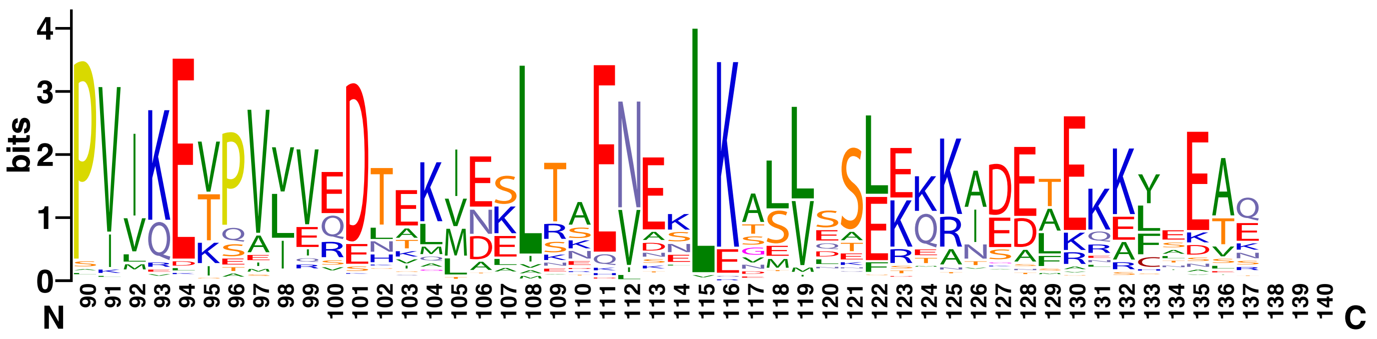

```
AtMyo11C1   960  PVIKETQILVEDTKKIELMTEELESVKVTLENEKQRADDAVRKFEEAQ---
AtMyo11C2   960  PVVTETQVLVEDTQKIEALTEEVEGLKANLEQEKQRADDATRKFDEAQ---
AtMyo11B1   959  PIIKEVPVV--DQELMEKLTNENEKLKGMVSSLEIKIDETAKELHETA---
AtMyo11B2   958  PIIKEIPVV--DQELMDKITNENEKLKSMVSSLEMKIGETEKKLQETT---
AtMyo11B3   957  PVLQEVPVV--DTELVEKLTSENEKLKSLVSSLDQKIDETEKKFEERS---
AtMyo11B4   953  PVVKEVPVV--DTVLMEKLTSENEKLKSLVTSLELKIDETEKKFEETK---
AtMyo11H    959  PIIKEVPVV--DNTQLELLNSQNNELEVEVAKLKGKIKEFEVKCFALE---
AtMyo11G    965  ISMKEKSAVERELNGMVELKKDNALLKNSMNSLEKKNRVLEKELLNAK---
AtMyo11E    960  PVIKETPVLVEDTEKINSLTSEVEALKASLQAERQAAENLRKAFSEAE---
AtMyo11F    957  SVNKE-PVVVEDTEKIDSLSNEIDRLKGLLSSETHKADEAQHAYQSAL---
```

C-terminal region 3

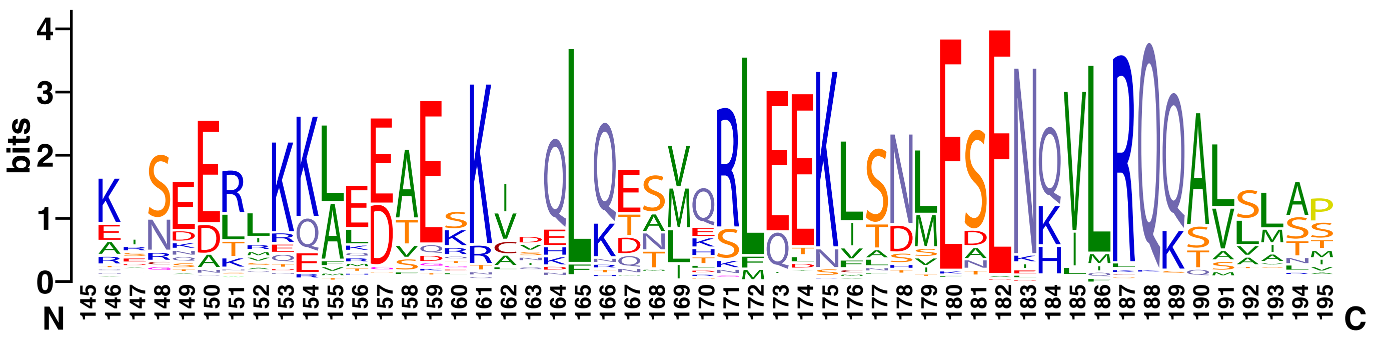

```
AtMyo11C1  1008  -ESLEDKKKKLEETEKKGQQLQESLTRMEEKCSNLESENKVLRQQAVSMAP
AtMyo11C2  1008  -ESSEDRKKKLEDTEKKAQQLQESVTRLEEKCNNLESENKVLRQQAVSIAP
AtMyo11B1  1005  -RISQDRLKQALAAESKVAKLKTAMQRLEEKISDMETEKQIMLQQT--ILN
AtMyo11B2  1004  -KISQDRLNQALEAESKLVKLKTAMQRLEEKILDMEAEKKIMHQQT--IS-
AtMyo11B3  1003  -KINEERLKQAIEAETTIVNLKTAVHELQEKILDVESENKILRQKS--LIQ
AtMyo11B4   999  -KISEERLKKALDAENKIDNLKTAMHNLEEKLKEVKLENNFLKES---VLT
AtMyo11H   1005  -NDSRASVTEAEDAKSKAVEFQEIIERLHTNLSNLESENQVLRQQALAA-S
AtMyo11G   1013  -TNCNNTLQKLKEAEKRCSELQTSVQSLEEKLSHLENENQVLMQKT--LIT
AtMyo11E   1008  -ARNSELATELENATRKADQLHESVQRLEEKLSNSESEIQVLRQQA--LAI
AtMyo11F   1004  -VQNEELCKKLEEAGRKIDQLQDSVQRFQEKVFSLESENKVLRQQT--LTI
```

C-terminal region 4

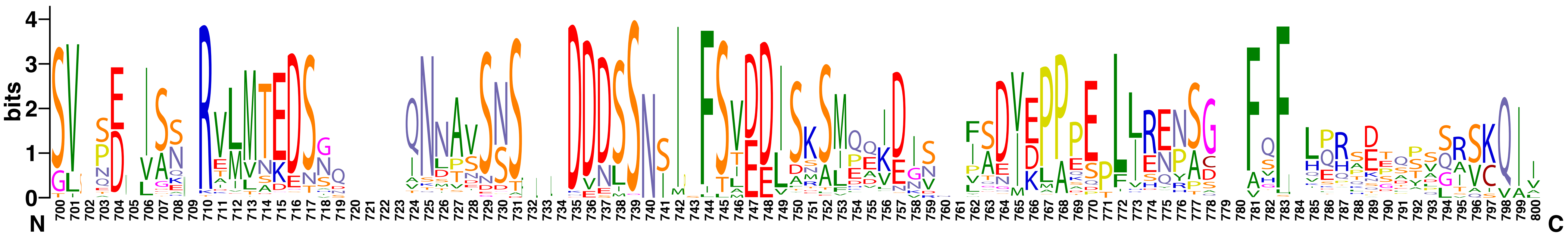

```
AtMyo11C1  1458  SVSPDVIANMRVLMTEDS-------NNAVSNSFLLDDDSS--IPFSVDDLSKSMEKFE----IADIEPPP--LIRENSG--FSFLLPVSE-----------
AtMyo11C2  1454  SVSPDVIANMRVLMTEDS-------NNAVSNSFLLDDDSS--IPFSVDDLSKSMERIE----IGDVEPPP--LIRENSG--FSFLLPCSD-----------
AtMyo11B1  1432  SVSQEVISSMRALMTEES-------NDADSNSFLLDDNSS--IPFSIDEISNSMHEKD----FASVKPAKE-LLENPE---FVFLH---------------
AtMyo11B2  1437  SVSQEVISSMRTLMTEES-------NDADSDSFLLDDDSS--IPFSIDDISSSMEEKD----FVGIKPAEE-LLENPA---FVFLH---------------
AtMyo11B3  1440  SVSQDVIANMRVLMTEDS-------NNADSSAFLLDEDSS--IPFSADDLSSSMKEKD----FAEMKPAEE-LEENPA---FSFLI---------------
AtMyo11B4  1418  SVSQDVIASMTGVMTDSS-------------DFLLKEDSSNIISLSIDDLCSSMQDKD----FAQVKPAEE-LLENPS---FIFLH---------------
AtMyo11H   1481  GLSPEVINQMRKLMTEDS------ANMTY-PSFLLDVDSS--IPFSVEDVSQSFHGGNIS--LSDVDPSP--LLRQRSD--FHFLFQTLPE----------
AtMyo11G   1441  SVSSEVVSQMRVLVDKDN------QKQTS-NSFLLDDDMS--IPFSAEDIDKAIPVLD----PSEIEPPK--FVSEYTC--AQSLVKKPSIAS--TSKQII
AtMyo11E   1455  SVSSDVIANMRVMMTEDS-------NNAVSSSFLLDDDSS--IPFTVEDISKSMQQVD----VNDIEPPQ--LIRENSG--FGFLLTRKEGSTS-------
AtMyo11F   1448  SVSTEVIATMRAEVSDVS-------KSAISNSFLLDDDSS--IPFSLDDISKSMQNVE----VAEVDPPP--LIRQNSN--FMFLLERSD-----------
```
