# Supplementary material for: Whole genome duplication events in plant evolution reconstructed and predicted using myosin motor proteins
Source: BMC Evol Biol. 2013 Sep 22;13:202. doi: 10.1186/1471-2148-13-202 (PMC3850447; doi:10.1186/1471-2148-13-202)
Supplement: Additional file 13 — Species and genome assembly information. This file contains the full taxonomy and the source of the genome assembly data for all species analysed. Genome analyses are referenced if these have already been published. All information is also available at CyMoBase (http://www.cymobase.org, [24]). [file 1471-2148-13-202-S13.pdf]

***Eutrema halophilum* phytozome assembly (Euh)**
Taxonomy:
cellular organisms | Eukaryota | Viridiplantae | Streptophyta | Streptophytina | Embryophyta | Tracheophyta | Euphyllophyta | Spermatophyta | Magnoliophyta | eudicotyledons | core eudicotyledons | rosids | malvids | Brassicales | Brassicaceae | Eutremeae | Eutrema
Reference/s:
Phytozome: Thellungiella halophila (Salt cress)
Yang R et. al., *Front Plant Sci* , **4** , 46 (2013).

***Eutrema halophilum* NCBI assembly (Ets)**
Taxonomy:
cellular organisms | Eukaryota | Viridiplantae | Streptophyta | Streptophytina | Embryophyta | Tracheophyta | Euphyllophyta | Spermatophyta | Magnoliophyta | eudicotyledons | core eudicotyledons | rosids | malvids | Brassicales | Brassicaceae | Eutremeae | Eutrema
Reference/s:
GenBank - NIH genetic sequence database: GenBank species TBLASTN WGS
International Species Sequencing Consortium: Thellungiella salsuginea Sequencing Consortium
Publication/s:
Wu HJ *et. al.* , *Proc Natl Acad Sci U S A* , **109** , 12219 (2012).

***Carica papaya* (Cip)**
Taxonomy:
cellular organisms | Eukaryota | Viridiplantae | Streptophyta | Streptophytina | Embryophyta | Tracheophyta | Euphyllophyta | Spermatophyta | Magnoliophyta | eudicotyledons | core eudicotyledons | rosids | malvids | Brassicales | Caricaceae | Carica
Reference/s:
ASGPB University of Hawaii at Manoa: The Hawaii Papaya Genome Project
GenBank - NIH genetic sequence database: GenBank species TBLASTN WGS
International Species Sequencing Consortium: Carica papaya Sequencing Consortium
Phytozome: Carica papaya (Papaya)
Publication/s:
Ming R *et. al.* , *Nature* , **452** , 991 (2008).

***Theobroma cacao* (Thc)**
Taxonomy:
cellular organisms | Eukaryota | Viridiplantae | Streptophyta | Streptophytina | Embryophyta | Tracheophyta | Euphyllophyta | Spermatophyta | Magnoliophyta | eudicotyledons | core eudicotyledons | rosids | malvids | Malvales | Malvaceae | Byttnerioideae | Theobroma
Reference/s:
Cacao Genome Database: Cacao Genome Sequencing
GenBank - NIH genetic sequence database: GenBank species TBLASTN
International Species Sequencing Consortium: Cacao Sequencing Consortium
The Gene Index Project: DFCI Theobroma cacao (Cocoa) Gene Index
Publication/s:
Argout X *et. al.* , *Nat Genet* , **43** , 101 (2011).
Jones PG *et. al.* , *Planta* , **216** , 255 (2002).

***Gossypium hirsutum* (Gh)**
Taxonomy:
cellular organisms | Eukaryota | Viridiplantae | Streptophyta | Streptophytina | Embryophyta | Tracheophyta | Euphyllophyta | Spermatophyta | Magnoliophyta | eudicotyledons | core eudicotyledons | rosids | malvids | Malvales | Malvaceae | Malvoideae | Gossypium

***Gossypium raimondii* (Gor)**
Taxonomy:
cellular organisms | Eukaryota | Viridiplantae | Streptophyta | Streptophytina | Embryophyta | Tracheophyta | Euphyllophyta | Spermatophyta | Magnoliophyta | eudicotyledons | core eudicotyledons | rosids | malvids | Malvales | Malvaceae | Malvoideae | Gossypium
Reference/s:
GenBank - NIH genetic sequence database: GenBank species TBLASTN WGS
International Species Sequencing Consortium: Gossypium raimondii Sequencing Consortium
Phytozome: Gossypium raimondii
Publication/s:
Paterson AH *et. al.* , *Nature* , **492** , 423 (2012).
Wang K *et. al.* , *Nat Genet* , **44** , 1098 (2012).

***Eucalyptus grandis* (Eug)**
Taxonomy:
cellular organisms | Eukaryota | Viridiplantae | Streptophyta | Streptophytina | Embryophyta | Tracheophyta | Euphyllophyta | Spermatophyta | Magnoliophyta | eudicotyledons | core eudicotyledons | rosids | malvids | Myrtales | Myrtaceae | Eucalyptus
Reference/s:
Phytozome: Eucalyptus grandis (Eucalyptus)

***Eucalyptus camaldulensis* (Euc)**
Taxonomy:
cellular organisms | Eukaryota | Viridiplantae | Streptophyta | Streptophytina | Embryophyta | Tracheophyta | Euphyllophyta | Spermatophyta | Magnoliophyta | eudicotyledons | core eudicotyledons | rosids | malvids | Myrtales | Myrtaceae | Eucalyptus
Reference/s:
GenBank - NIH genetic sequence database: GenBank species TBLASTN WGS
International Species Sequencing Consortium: Eucalyptus camaldulensis Sequencing Consortium
Kazusa DNA Research Institute: Eucalyptus camaldulensis Genome Database
Publication/s:
Hirakawa H *et. al.* , *Plant Biotechnol* , **28** , 471 (2011).

***Citrus sinensis* (Cts)**
Taxonomy:
cellular organisms | Eukaryota | Viridiplantae | Streptophyta | Streptophytina | Embryophyta | Tracheophyta | Euphyllophyta | Spermatophyta | Magnoliophyta | eudicotyledons | core eudicotyledons | rosids | malvids | Sapindales | Rutaceae | Citrus
Reference/s:
GenBank - NIH genetic sequence database: GenBank species TBLASTN WGS
International Species Sequencing Consortium: Citrus sinensis Sequencing Consortium
Phytozome: Citrus sinensis (Sweet orange)
Publication/s:
Xu Q *et. al.* , *Nat Genet* , **45** , 59 (2012).

***Citrus clementina* (Cic)**
Taxonomy:
cellular organisms | Eukaryota | Viridiplantae | Streptophyta | Streptophytina | Embryophyta | Tracheophyta | Euphyllophyta | Spermatophyta | Magnoliophyta |
