# Supplementary material for: Whole genome duplication events in plant evolution reconstructed and predicted using myosin motor proteins
Source: BMC Evol Biol. 2013 Sep 22;13:202. doi: 10.1186/1471-2148-13-202 (PMC3850447; doi:10.1186/1471-2148-13-202)
Supplement: Additional file 3 — Phylogenetic trees of the plant myosins based on the motor domain and full-length sequences. The grouping of plant myosin sequences into different subtypes is based on the phylogenetic trees calculated with RAxML (bootstrap values in percentages), MrBayes (posterior probabilities) and ClustalW (bootstrap values as absolute occurrences of branchings in 1,000 trees). Different subsets of the myosin sequences (the alignment is available in Additional file 8) were used to calculated the trees: the myosin head domains of representative sequences with less than 90% sequence identity (as obtained by CD-Hit), the myosin head domains of all complete myosin sequences included in this analysis, and the full-length sequences of representative sequences below an 90% identity threshold (CD-Hit). All resulting trees are included in this file. The protein abbreviation “Myo” was omitted in phylogenetic trees due to length limitations of sequence names in tree reconstruction software. [file 1471-2148-13-202-S3.pdf]

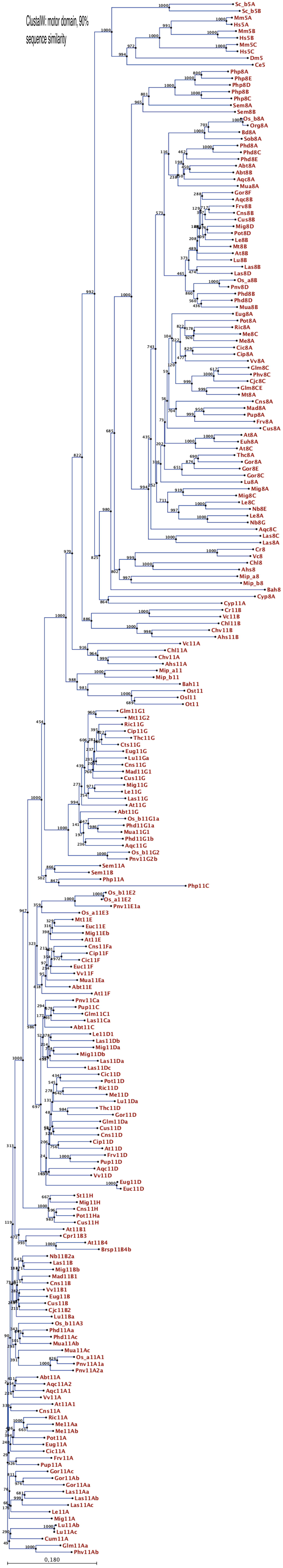
ClustalW: motor domain, 90%
sequence similarity
0,180

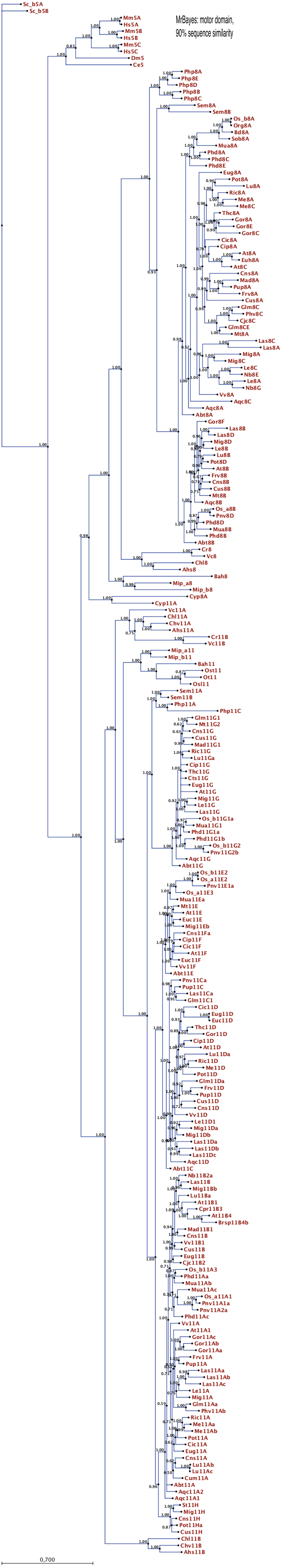
MrBayes: motor domain, 90% sequence similarity
Sc_b5A
Sc_b5B
Mm5A
Hs5A
Mm5B
Hs5B
Mm5C
Hs5C
Dm5
Ce5
Php8A
Php8E
Php8D
Php8B
Php8C
Sem8A
Sem8B
Os_b8A
Org8A
Bd8A
Sob8A
Mua8A
Phd8A
Phd8C
Phd8E
Eug8A
Pot8A
Lu8A
Ric8A
Me8A
Me8C
Thc8A
Gor8A
Gor8E
Gor8C
Cic8A
Cip8A
At8A
Euh8A
At8C
Cns8A
Mad8A
Pup8A
Frv8A
Cus8A
Glm8C
Phv8C
Cjc8C
Glm8CE
Mt8A
Las8C
Las8A
Mig8A
Mig8C
Le8C
Nb8E
Le8A
Nb8G
Vv8A
Aqc8C
Aqc8A
Abt8A
Gor8F
Las8B
Las8D
Mig8D
Le8B
Lu8B
Pot8D
At8B
Frv8B
Cns8B
Cus8B
Mt8B
Aqc8B
Os_a8B
Pnv8D
Phd8D
Mua8B
Phd8B
Abt8B
Cr8
Vc8
Chl8
Ahs8
Bah8
Mip_a8
Mip_b8
Cyp8A
Cyp11A
Vc11A
Chl11A
Chv11A
Ahs11A
Cr11B
Vc11B
Mip_a11
Mip_b11
Bah11
Ost11
Ot11
Osl11
Sem11A
Sem11B
Php11A
Php11C
Glm11G1
Mt11G2
Cns11G
Cus11G
Mad11G1
Ric11G
Lu11Ga
Cip11G
Thc11G
Cts11G
Eug11G
At11G
Mig11G
Le11G
Las11G
Os_b11G1a
Mua11G1
Phd11G1a
Phd11G1b
Os_b11G2
Pnv11G2b
Aqc11G
Abt11G
Os_b11E2
Os_a11E2
Pnv11E1a
Os_a11E3
Mua11Ea
Mt11E
At11E
Euc11E
Mig11Eb
Cns11Fa
Cip11F
Cic11F
At11F
Euc11F
Vv11F
Abt11E
Pnv11Ca
Pup11C
Las11Ca
Glm11C1
Cic11D
Eug11D
Euc11D
Thc11D
Gor11D
Cip11D
At11D
Lu11Da
Ric11D
Me11D
Pot11D
Glm11Da
Frv11D
Pup11D
Cus11D
Cns11D
Vv11D
Le11D1
Mig11Da
Mig11Db
Las11Da
Las11Db
Las11Dc
Aqc11D
Abt11C
Nb11B2a
Las11B
Mig11Bb
Lu11Ba
At11B1
Cpr11B3
At11B4
Brsp11B4b
Mad11B1
Cns11B
Vv11B1
Cus11B
Eug11B
Cjc11B2
Os_b11A3
Phd11Aa
Mua11Ab
Mua11Ac
Os_a11A1
Pnv11A1a
Pnv11A2a
Phd11Ac
Vv11A
At11A1
Gor11Ac
Gor11Ab
Gor11Aa
Frv11A
Pup11A
Las11Aa
Las11Ab
Las11Ac
Le11A
Mig11A
Glm11Aa
Phv11Ab
Ric11A
Me11Aa
Me11Ab
Pot11A
Cic11A
Eug11A
Cns11A
Lu11Ab
Lu11Ac
Cum11A
Abt11A
Aqc11A2
Aqc11A1
St11H
Mig11H
Cns11H
Pot11Ha
Cus11H
Chl11B
Chv11B
Ahs11B
0,700

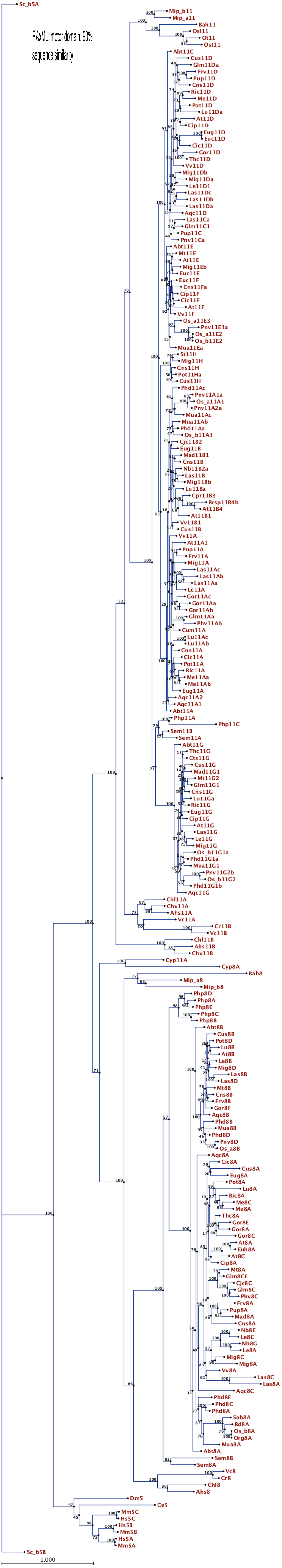
RAxML: motor domain, 90% sequence similarity
Sc_b5A
Mip_b11
Mip_a11
Bah11
Osl11
Ot11
Ost11
Abt11C
Cus11D
Glm11Da
Frv11D
Pup11D
Cns11D
Ric11D
Me11D
Pot11D
Lu11Da
At11D
Cip11D
Eug11D
Euc11D
Cic11D
Gor11D
Thc11D
Vv11D
Mig11Db
Mig11Da
Le11D1
Las11Dc
Las11Db
Las11Da
Aqc11D
Las11Ca
Glm11C1
Pup11C
Pnv11Ca
Abt11E
Mt11E
At11E
Mig11Eb
Euc11E
Euc11F
Cns11Fa
Cip11F
Cic11F
At11F
Vv11F
Os_a11E3
Pnv11E1a
Os_a11E2
Os_b11E2
Mua11Ea
St11H
Mig11H
Cns11H
Pot11Ha
Cus11H
Phd11Ac
Pnv11A1a
Os_a11A1
Pnv11A2a
Mua11Ac
Mua11Ab
Phd11Aa
Os_b11A3
Cjc11B2
Eug11B
Mad11B1
Cns11B
Nb11B2a
Las11B
Mig11Bb
Lu11Ba
Cpr11B3
Brsp11B4b
At11B4
At11B1
Vv11B1
Cus11B
Vv11A
At11A1
Pup11A
Frv11A
Mig11A
Las11Ac
Las11Ab
Las11Aa
Le11A
Gor11Ac
Gor11Aa
Gor11Ab
Glm11Aa
Phv11Ab
Cum11A
Lu11Ac
Lu11Ab
Cns11A
Cic11A
Pot11A
Ric11A
Me11Aa
Me11Ab
Eug11A
Aqc11A2
Aqc11A1
Abt11A
Php11A
Php11C
Sem11B
Sem11A
Abt11G
Thc11G
Cts11G
Cus11G
Mad11G1
Mt11G2
Glm11G1
Cns11G
Lu11Ga
Ric11G
Eug11G
Cip11G
At11G
Las11G
Le11G
Mig11G
Os_b11G1a
Phd11G1a
Mua11G1
Pnv11G2b
Os_b11G2
Phd11G1b
Aqc11G
Chl11A
Chv11A
Ahs11A
Vc11A
Cr11B
Vc11B
Chl11B
Ahs11B
Chv11B
Cyp11A
Cyp8A
Bah8
Mip_a8
Mip_b8
Php8D
Php8A
Php8E
Php8C
Php8B
Abt8B
Cus8B
Pot8D
Lu8B
At8B
Le8B
Mig8D
Las8B
Las8D
Mt8B
Cns8B
Frv8B
Gor8F
Aqc8B
Phd8B
Mua8B
Phd8D
Pnv8D
Os_a8B
Aqc8A
Cic8A
Cus8A
Eug8A
Pot8A
Lu8A
Ric8A
Me8C
Me8A
Thc8A
Gor8E
Gor8A
Gor8C
At8A
Euh8A
At8C
Cip8A
Mt8A
Glm8CE
Cjc8C
Glm8C
Phv8C
Frv8A
Pup8A
Mad8A
Cns8A
Nb8E
Le8C
Nb8G
Le8A
Mig8C
Mig8A
Vv8A
Las8C
Las8A
Aqc8C
Phd8E
Phd8C
Phd8A
Sob8A
Bd8A
Os_b8A
Org8A
Mua8A
Abt8A
Sem8B
Sem8A
Vc8
Cr8
Chl8
Ahs8
Dm5
Ce5
Mm5C
Hs5C
Hs5B
Mm5B
Hs5A
Mm5A
Sc_b5B
1,000

ClustalW: motor domain, all sequences

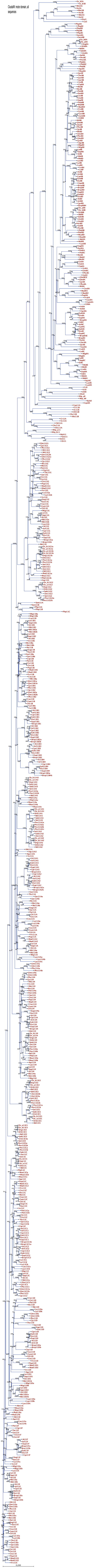

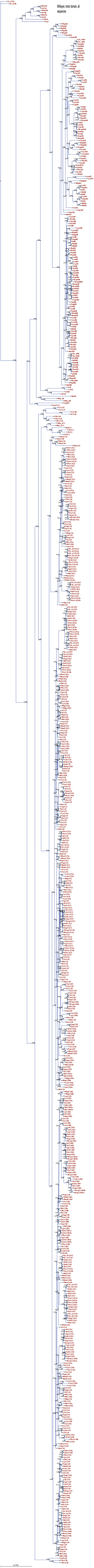
MrBayes: motor domain, all sequences

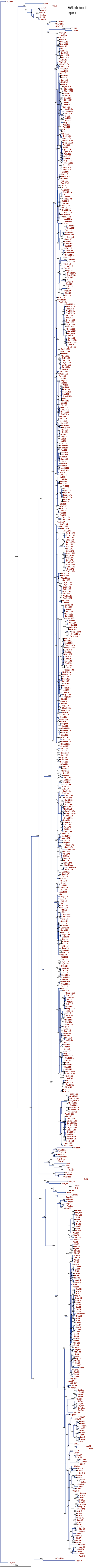
RAxML: motor domain, all sequences

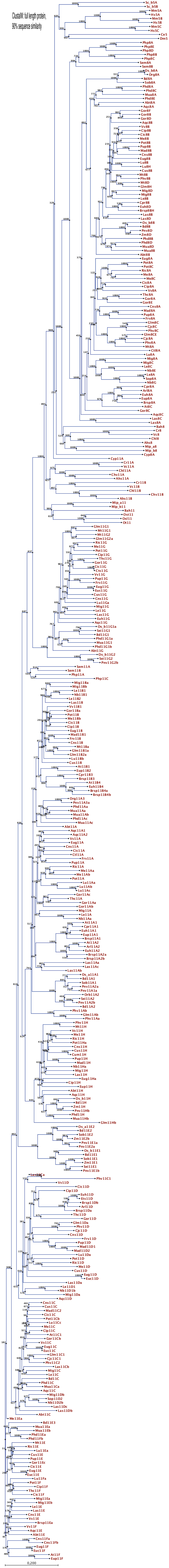
ClustalW: full length protein,
90% sequence similarity
0.200

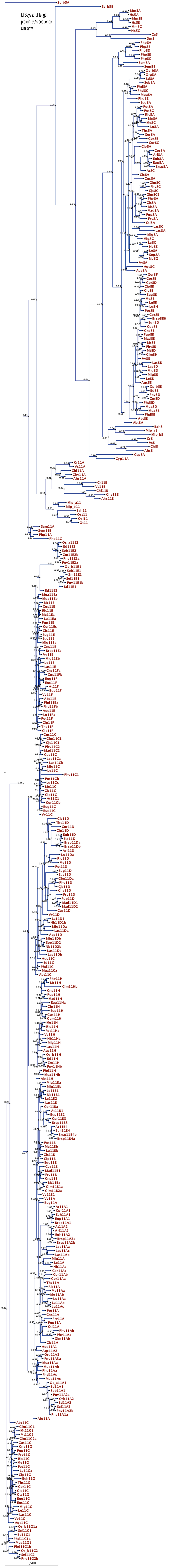
MrBayes: full length protein, 90% sequence similarity
1.500

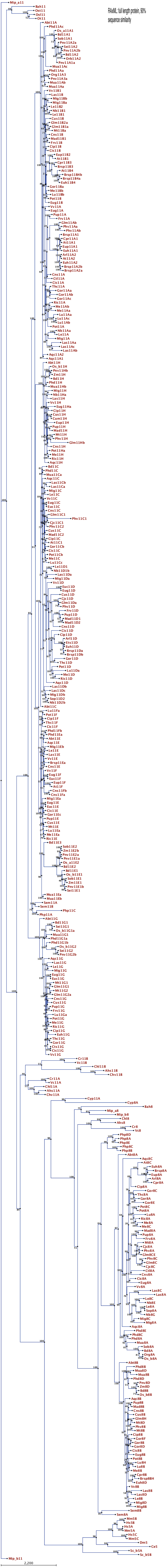
RAxML: full length protein, 90% sequence similarity
2.200
